# Harpagophytum procumbens in musculoskeletal disorders: current evidence and comparison with NSAIDs

**DOI:** 10.3389/fphar.2026.1839470

**Published:** 2026-06-17

**Authors:** Jin Young Hong, Junseon Lee, Hyunseong Kim, Hyun Kim, Wan-Jin Jeon, Changhwan Yeo, Yoon Jae Lee, Ho-Yeon Go, In-Hyuk Ha

**Affiliations:** 1 Jaseng Spine and Joint Research Institute, Jaseng Medical Foundation, Seoul, Republic of Korea; 2 Department of Korean Internal Medicine, College of Korean Medicine, Semyung University, Jecheon-si, Republic of Korea

**Keywords:** alternative, devil’s claw, Harpagophytum procumbens, inflammation, NSAIDs

## Abstract

Harpagophytum procumbens (HP), commonly known as Devil’s Claw, is a traditional medicinal plant widely used for musculoskeletal pain and inflammatory conditions. Interest in HP has increased because long-term use of non-steroidal anti-inflammatory drugs (NSAIDs) may be limited by gastrointestinal, cardiovascular, and renal adverse effects. This structured narrative review summarizes current evidence regarding the pharmacological mechanisms, clinical efficacy, safety, pharmacokinetics, regulatory status, and comparative role of HP relative to NSAIDs. Preclinical studies suggest that HP and its constituents, particularly harpagoside, may modulate inflammatory pathways including cyclooxygenase-2, nuclear factor-κB, pro-inflammatory cytokines, and oxidative stress signaling. However, these mechanistic findings represent indirect evidence and should be interpreted cautiously in relation to clinical outcomes. Clinical studies, mainly in osteoarthritis and low back pain, suggest that selected HP preparations may provide symptomatic benefit in some patients. However, the clinical evidence base remains limited by small sample sizes, heterogeneous formulations, variable dosages, short follow-up durations, and a scarcity of high-quality head-to-head randomized trials against NSAIDs. In contrast, NSAIDs are supported by a substantially broader and higher-certainty evidence base for pain relief and functional improvement. Available safety data suggest that HP is generally well tolerated, although product quality, standardization, and long-term safety data remain variable across jurisdictions. Current evidence does not support positioning HP as a general replacement for NSAIDs. Rather, HP may be considered as a complementary or selective option for some patients, particularly where NSAID intolerance or long-term safety concerns exist. Further large-scale, rigorously designed randomized trials using standardized formulations are required.

## 1 Introduction

Musculoskeletal disorders are characterized not only by chronic inflammation and pain but also by oxidative stress, nociceptive sensitization, and progressive tissue degeneration. These interconnected mechanisms contribute to persistent pain, functional impairment, and structural damage, thereby providing a rationale for therapeutic approaches targeting multiple inflammatory and cellular pathways (Bonanni et al., 2022; Puntillo et al., 2021).

Chronic inflammatory disorders such as osteoarthritis, rheumatoid arthritis, and low back pain are among the most prevalent causes of pain and disability worldwide (Verhaar et al., 2021). Non-steroidal anti-inflammatory drugs (NSAIDs) are widely used as first-line pharmacological agents for treatment in these conditions due to their rapid and effective symptom control (Ghlichloo and Gerriets, 2026). However, long-term NSAID use is frequently limited by adverse effects on the gastrointestinal, cardiovascular, and renal systems, prompting growing interest in safer, plant-based alternatives with comparable efficacy (Bindu et al., 2020).

*Harpagophytum procumbens* (HP) is a traditional medicinal plant native to Southern Africa that has been used for centuries for its anti-inflammatory and analgesic properties. Currently, it is also used in Korea by Oriental medicine doctors as a herbal medicine called *Cheonsugeon* for the management of conditions such as herniated intervertebral disc and arthritis. Its pharmacological activity is primarily attributed to harpagoside, an iridoid glycoside known to inhibit key inflammatory mediators such as Cyclooxygenase-2 (COX-2), iNOS, and NF-κB (Gxaba and Manganyi, 2022). These mechanistic findings have generated interest in HP as a potential complementary therapeutic option for musculoskeletal disorders. In addition to musculoskeletal applications, HP has also been reported to exhibit broader pharmacological activities in experimental cardiovascular, metabolic, neurological, and antimicrobial models (Boje et al., 2003; Chen et al., 2018; Chung et al., 2017; Circosta et al., 1984; Clarkson et al., 2003; Costa De Pasquale et al., 1985; Ferrante et al., 2017; Kim et al., 2015; Lall et al., 2019; Lang and Xiong, 2025; Mahomed and Ojewole, 2004; Mahomed and Ojewole, 2006; Manon et al., 2015; Modarai et al., 2011; Occhiuto et al., 1985; Quarta et al., 2022; Radomska-Leśniewska et al., 2016; Shirzad et al., 2024; Torres-Fuentes et al., 2014; Weckesser et al., 2007; Mahomed and Ojewole, 2009; Ali et al., 2018).

Although numerous experimental and clinical studies have investigated the anti-inflammatory and analgesic properties of HP, the currently available evidence remains heterogeneous with respect to extract formulations, harpagoside content, dosage regimens, study populations, and clinical outcome measures. Furthermore, direct comparative clinical studies between HP and conventional NSAID therapies remain relatively limited. Consequently, interpretation of comparative efficacy and safety between HP and NSAIDs requires cautious and balanced evaluation.

Accordingly, this structured narrative review critically examines the currently available preclinical and clinical evidence regarding HP in comparison with NSAIDs. Particular emphasis is placed on comparative efficacy, analgesic and anti-inflammatory mechanisms, safety and tolerability profiles, pharmacokinetic characteristics, regulatory considerations, and current limitations of the evidence base.

### 1.1 Methods

**SCHEME 1:**
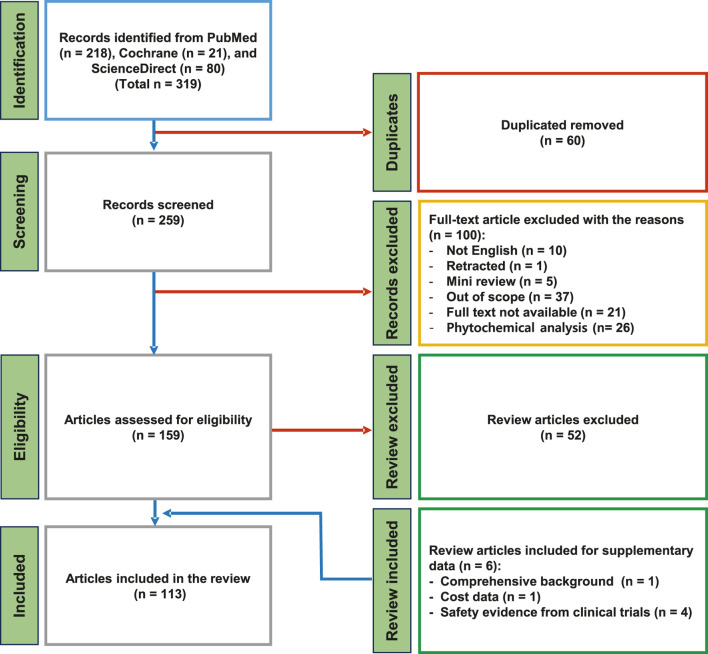
Overview of the literature search, screening, categorization, and narrative synthesis process used in this structured narrative review of *Harpagophytum procumbens* and NSAIDs.

This manuscript was conducted as a structured narrative review rather than a formal systematic review. A targeted literature search was performed using PubMed, Cochrane Library, and ScienceDirect to identify relevant English-language publications addressing Harpagophytum procumbens in relation to musculoskeletal disorders, inflammation, pain, safety, pharmacology, and comparison with NSAIDs. Search terms included combinations of “Harpagophytum procumbens”, “Devil’s Claw”, “musculoskeletal pain”, “osteoarthritis”, “low back pain”, “arthritis”, “NSAIDs”, “clinical trial”, and “safety”. Reference lists of relevant articles were also screened to identify additional studies. Eligible sources included clinical trials, observational studies, systematic reviews, pharmacological studies, and selected preclinical investigations considered relevant to the review objectives. Conference abstracts, commentaries, duplicate publications, and studies lacking sufficient methodological detail were excluded. Two reviewers independently screened titles and abstracts, followed by full-text review of potentially relevant articles. Data were extracted on study design, population, intervention characteristics, comparator treatments, outcomes, and safety findings. Because of substantial heterogeneity in formulations, dosages, outcome measures, and study designs, findings were synthesized narratively rather than quantitatively. No formal meta-analysis was undertaken. The literature identification and thematic selection process is summarized in Scheme 1. The figure illustrates how potentially relevant records were screened and grouped to support the narrative synthesis rather than a formal PRISMA-style systematic review selection process.

## 2 Preclinical pharmacology of HP

### 2.1 Anti-inflammatory effects

Detailed *in vitro* and *ex vivo* anti-inflammatory studies are summarized in Supplementary Table S1, and *in vivo* animal studies are summarized in Supplementary Table S2. Preclinical studies suggest that HP possesses anti-inflammatory activity across *in vitro*, *ex vivo*, and animal models, with harpagoside, harpagide, and leucosceptoside A frequently identified as relevant bioactive constituents. In cellular systems, HP extracts and isolated compounds have been reported to reduce inflammatory mediators including COX-1/2, PGE2, TNF-α, IL-1β, IL-6, nitric oxide, and related markers in macrophages, monocytes, chondrocytes, synoviocytes, microglia, and other immune or joint-relevant models (Quarta et al., 2022; Anauate et al., 2010; Fiebich et al., 2001; Fiebich et al., 2012; Günther et al., 2006; Gyurkovska et al., 2011; Haseeb et al., 2017; Hostanska et al., 2014; Huang et al., 2006; Inaba et al., 2010; Jang et al., 2003; Kaszkin et al., 2004; Kim and Park, 2015; Koycheva et al., 2021; Mariano et al., 2024; Mariano et al., 2022; Mariano et al., 2020; Na et al., 2004; Schopohl et al., 2016; Ungerer et al., 2020). These effects were accompanied by modulation of NF-κB, MAPKs, PI3K/Akt, AP-1, and c-Fos signaling pathways, suggesting multi-target anti-inflammatory activity.

Comparative evidence against NSAID controls remains limited but informative. In the only identified direct *in vitro* comparison, Anauate et al. used LPS-stimulated human whole blood and evaluated three HP fractions differing in harpagoside content (Anauate et al., 2010). Indomethacin and etoricoxib produced substantially greater inhibition of COX-related pathways, whereas the most active HP fraction, containing the highest harpagoside concentration, demonstrated moderate but significant COX-1 and COX-2 inhibition. Fractions with lower harpagoside content showed minimal activity, indicating that extract composition may be a key determinant of anti-inflammatory potency.

*Ex vivo* studies also supported anti-inflammatory potential. In porcine skin models, HP extracts and selected glycosides reduced COX-2 and PGE2 expression, with some compounds demonstrating responses comparable to ibuprofen under specific experimental conditions (Ouitas and Heard, 2009; Abdelouahab N. and Heard C., 2008; Abdelouahab N. and Heard CM., 2008). In rat colon tissue, microwave-assisted HP extracts suppressed PGE2, 5-HT, and TNF-α production, with activity comparable to sulfasalazine in that model (Locatelli et al., 2017). Additional murine colon studies demonstrated reductions in NF-κB, TNF-α, and IL-6 expression (Recinella et al., 2020).

Animal studies demonstrated reductions in edema, inflammatory biomarkers, and arthritis-related swelling across acute and chronic models (Mahomed and Ojewole, 2004; Inaba et al., 2010; Na et al., 2004; Andersen et al., 2004; Baghdikian et al., 1997; Catelan et al., 2006; Kundu et al., 2005; Lanhers et al., 1992; Peruru et al., 2020; Wachsmuth et al., 2011; Soulimani et al., 1994; Chrubasik et al., 2006). In carrageenan-induced paw edema, high-dose HP extract achieved inhibition approaching that reported for indomethacin in some experiments (Baghdikian et al., 1997; Lanhers et al., 1992), whereas in egg albumin-induced edema, efficacy was lower than diclofenac (Mahomed and Ojewole, 2004). Other studies reported reductions in CRP, cytokines, and leukocyte counts, although osteoarthritis structural outcomes were less consistent and not always statistically significant (Inaba et al., 2010; Catelan et al., 2006; Peruru et al., 2020).

Overall, available preclinical evidence supports biologically plausible anti-inflammatory activity of HP. However, heterogeneous extracts, variable constituent content, diverse dosing strategies, surrogate endpoints, and non-clinical models limit direct clinical interpretation. Notably, comparative findings with NSAIDs were inconsistent across experimental models. For example, in carrageenan-induced paw edema models, high-dose HP extracts achieved inhibition approaching that of indomethacin (Baghdikian et al., 1997; Lanhers et al., 1992), whereas in egg albumin-induced edema models, efficacy was lower than that of diclofenac (Mahomed and Ojewole, 2004). Such model-dependent variability suggests that the anti-inflammatory effects of HP are not consistently comparable to those of NSAIDs, and reliable prediction of NSAID-equivalent efficacy remains uncertain.

### 2.2 Analgesic (antinociceptive) effects

The antinociceptive effects of HP have been investigated primarily in preclinical *in vivo* studies, as summarized in Supplementary Table S3. Most currently available evidence regarding the analgesic effects of HP has been derived from animal models, and the clinical relevance of these findings in human pain conditions remains incompletely established.

Traditionally, HP has been used for the management of inflammatory pain, and several experimental studies have suggested potential antinociceptive activity in inflammatory pain models. Lanhers et al. reported that water extracts of HP produced dose-dependent reductions in acetic acid-induced writhing responses and thermal nociception in animal models. HP extracts administered at 100 and 400 mg/kg reportedly produced 47% and 78% protection against writhing responses, respectively (Lanhers et al., 1992). The observed effects were considered comparable under the tested experimental conditions to those produced by acetylsalicylic acid (68 mg/kg) and morphine sulfate (1.15 mg/kg), although direct translational comparisons between these agents remain difficult because of differences in pharmacological mechanisms and dosing paradigms. Similarly, Baghdikian et al. reported that HP extract (1,200 mg/kg) reduced acetic acid-induced writhing responses, with effects observed under the specific experimental conditions appearing similar to those reported for acetylsalicylic acid (Baghdikian et al., 1997). Mahomed et al. further demonstrated analgesic effects of aqueous HP extracts (50–800 mg/kg) in chemical and thermal nociception models, while also reporting an LD_50_ value of 1,250 ± 156 mg/kg, suggesting relatively low acute toxicity under the tested experimental conditions (Mahomed and Ojewole, 2004).

Several studies have also explored the potential effects of HP in neuropathic and postoperative pain models. Lim et al. evaluated HP in plantar incision and spared nerve injury (SNI) models and reported that oral administration of HP (300 mg/kg) restored mechanical withdrawal thresholds and reduced ultrasonic vocalizations associated with affective pain behaviors (Lim et al., 2014). In the plantar incision model, the observed analgesic effects of HP under the experimental conditions appeared comparable to those reported for naproxen (30 mg/kg). HP also reduced 22–27 kHz ultrasonic vocalizations by 73.5% (p < 0.01), suggesting potential modulation of both behavioral and affective pain responses. In the SNI model, HP administration progressively attenuated pain sensitivity from days 3–21 post-surgery, with an 83.6% recovery in mechanical withdrawal threshold by Day 21 (p < 0.001). These findings may suggest potential activity of HP in neuropathic pain-related pathways.

Potential interactions between HP and opioid-related analgesic pathways have also been investigated in experimental studies. Parenti et al. (2015) reported that HP extract reduced mechanical allodynia and thermal hyperalgesia in a carrageenan-induced inflammatory pain model, with the observed effects potentially involving activation of the heme oxygenase-1 (HO-1)/carbon monoxide (CO) pathway (Parenti et al., 2015). The analgesic effects were attenuated by ZnPP IX, an HO-1 inhibitor, and appeared enhanced when combined with hemin or CORM-3. In a subsequent chronic constriction injury (CCI) study, Parenti et al. (2016) reported that co-administration of HP (400 mg/kg) and morphine (3 mg/kg) produced greater analgesic responses than either treatment alone under the experimental conditions evaluated (Parenti et al., 2016). Although these findings may suggest the possibility of adjunctive interactions with opioid analgesics, the clinical relevance and safety implications of such combinations remain unknown.

Additional mechanistic studies have suggested that multiple signaling pathways may contribute to the observed antinociceptive effects of HP. Uchida et al. reported that HP administration significantly reduced both phases of formalin-induced nociceptive responses, and these effects were partially reversed by naloxone, suggesting possible involvement of opioid-related pathways (Uchida et al., 2008). The same study also reported reductions in spinal nitric oxide metabolites, indicating potential modulation of nitric oxide-related signaling. Andersen et al. demonstrated analgesic effects of HP in a Freund’s adjuvant-induced arthritis model, with improvements observed in joint swelling and pain thresholds (Andersen et al., 2004). More recently, Hong et al. reported that HP reduced TRPV1 expression in dorsal root ganglion neurons and increased withdrawal latency in a lumbar spinal stenosis model, suggesting possible modulation of TRPV1-associated nociceptive pathways (Hong et al., 2022).

HP preparations may exert antinociceptive effects across inflammatory, postoperative, and neuropathic pain models. The reported analgesic activities appear to involve multiple pathways related to inflammatory signaling, oxidative stress modulation, opioid-related mechanisms, nitric oxide regulation, and TRPV1-associated nociceptive processing. In particular, several experimental studies have suggested potential effects of HP on both sensory and affective components of pain, including mechanical hypersensitivity and pain-associated behavioral responses. However, the available evidence remains predominantly preclinical, and relatively few studies have included standardized positive comparators such as conventional NSAIDs or opioid analgesics. In addition, variations in extract composition, dosing regimens, and experimental methodologies complicate direct comparison across studies. Therefore, although HP demonstrates potential antinociceptive activity in experimental settings, current evidence remains insufficient to establish HP as a definitive alternative to conventional analgesic therapies.

### 2.3 Antioxidant effects

Evidence regarding the antioxidant properties of HP has been evaluated across multiple cellular, tissue, and animal models, with detailed experimental findings summarized in Supplementary Tables S4, S5.

Preclinical studies suggest that HP exhibits antioxidant activity across cellular and tissue models, primarily through modulation of oxidative stress pathways and activation of endogenous antioxidant defenses. *In vitro* and *ex vivo* studies consistently report reductions in reactive oxygen species (ROS), nitric oxide, and lipid peroxidation markers, together with activation of NRF2/HO-1 signaling and suppression of pro-oxidant enzymes such as COX-2 and iNOS (Quarta et al., 2022; Huang et al., 2006; Ungerer et al., 2020; Locatelli et al., 2017; Soulimani et al., 1994; Chrubasik et al., 2006; Lim et al., 2014; Parenti et al., 2015). These findings support a biologically plausible role of HP in redox regulation, although most evidence is derived from surrogate biochemical endpoints.

Direct comparative antioxidant data are relatively sparse but provides some mechanistic insight. In a spinal cord contusion injury model, HP administration (300 mg/kg) reduced lipid peroxidation markers (4-HNE), restored 4-HHE levels, and increased HO-1 expression, with oxidative stress responses reported to be similar to those observed with ibuprofen (20 mg/kg) under the same experimental conditions (Ungerer et al., 2020). In other experimental settings, antioxidant effects of HP were evaluated using different reference compounds (e.g., Trolox, gallic acid, apocynin), but these comparisons relied on distinct endpoints such as radical scavenging activity, lipid peroxidation indices, or modulation of antioxidant enzymes across heterogeneous biological systems (Recinella et al., 2020; Peruru et al., 2020; Schaffer et al., 2013). As a result, these findings represent context-specific biochemical effects rather than standardized or directly comparable measures of antioxidant efficacy.

Additional *in vivo* studies without NSAID comparators demonstrated reductions in oxidative stress markers (e.g., MDA, NO, nitrite/nitrate) and increases in antioxidant defenses (e.g., GSH, SOD, catalase) across various inflammatory and neurotoxic models (Peruru et al., 2020; Parenti et al., 2015; Schaffer et al., 2013; Calabrese et al., 2021; Grant et al., 2009; Langmead et al., 2002; Lima et al., 2023). However, these studies were generally exploratory, employed diverse formulations or combination products, and used heterogeneous outcome measures, limiting cross-study comparability.

Taken together, HP demonstrates promising antioxidant properties in experimental systems. Nevertheless, current evidence is based largely on heterogeneous laboratory models and surrogate oxidative stress markers, and the pathophysiological relevance of these findings to musculoskeletal disorders remains incompletely defined.

### 2.4 Cytoprotective effects

The cytoprotective properties of HP have been investigated across a range of *in vitro* models, primarily focusing on cell viability and resistance to oxidative or inflammatory injury (Supplementary Table S6). In several cell systems, including fibroblasts, macrophages, neutrophils, renal cells, and myogenic cells, HP extracts demonstrated minimal cytotoxicity, with cell viability generally maintained above 85%–90% across concentrations up to approximately 1 mg/mL under short-term exposure conditions (Chung et al., 2017; Jang et al., 2003; Grant et al., 2009; Chung et al., 2016).

However, evidence from more sensitive cell models indicates that these effects are not uniformly preserved. For example, in BV-2 microglial cells, HP at 300 μg/mL reduced cell viability to below 50% following 72-h exposure (Lima et al., 2023), while in neuronal Hypo-E22 cells, significant reductions in viability were observed at concentrations of 100–200 μg/mL (Ferrante et al., 2017). These findings indicate that cytoprotective effects of HP are dose- and cell-type-dependent, with a narrower therapeutic margin in neuronal systems.

Neuroprotective effects have been reported in models of oxidative and excitotoxic injury. HP treatment improved cell viability and reduced necrotic cell death in neuronal systems exposed to oxidative stressors, including iron-induced injury and nitric oxide donors (Ferrante et al., 2017; Hong et al., 2022; Schaffer et al., 2013). These effects were associated with modulation of oxidative stress pathways and anti-apoptotic mechanisms. However, protective responses were not consistently observed across all concentrations, and higher doses were occasionally associated with reduced viability.

Additional cytoprotective findings have been reported in bone-related and other tissue models. HP and its constituents showed minimal cytotoxicity in osteoblast and osteoclast precursor cells and did not impair differentiation processes at tested concentrations (Chung et al., 2017; Kim et al., 2015; Chung et al., 2016). In contrast, cytotoxic effects were observed in certain cancer cell lines at high concentrations, suggesting that HP may exert context-dependent effects depending on cell type and experimental conditions (Locatelli et al., 2017; Recinella et al., 2020).

Current evidence indicates that cytoprotective effects of HP are context-dependent, varying according to cell type, exposure conditions, and experimental model. While protective responses are frequently observed in immune and connective tissue-related systems, they are less consistent in neuronal models and at higher exposure levels, suggesting limited generalizability across experimental settings.

## 3 Comparative evaluation of HP and NSAIDs

### 3.1 Mechanisms of action vs. NSAIDs

NSAIDs are widely used pharmacological agents for the treatment of pain and inflammation in musculoskeletal disorders. Their therapeutic effects are primarily mediated through inhibition of COX enzymes, particularly COX-2, resulting in suppression of prostaglandin synthesis. Owing to their relatively well-defined mechanisms and extensive validation in randomized controlled trials, NSAIDs remain a standard therapeutic option across a broad range of musculoskeletal conditions. However, prolonged systemic use is associated with well-recognized gastrointestinal, renal, and cardiovascular adverse effects (Qureshi and Dua, 2026).

**FIGURE 1:**
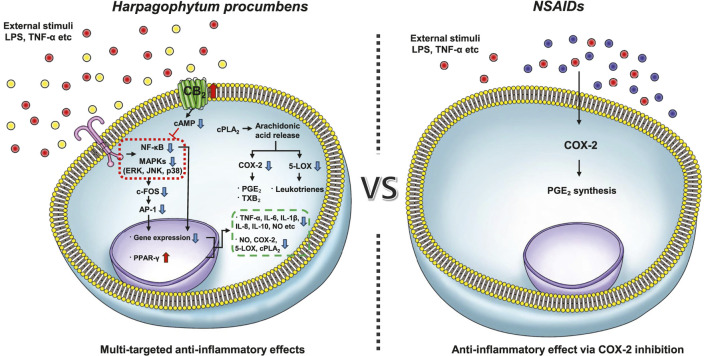
Schematic representation of the anti-inflammatory mechanisms of *HP* compared to NSAIDs. HP exerts anti-inflammatory effects through multiple pathways, including inhibition of MAPKs and NF-κB signaling, activation of PPAR-γ, and suppression of pro-inflammatory mediators (e.g., cytokines, COX-2, 5-LOX, cPLA_2_). In contrast, NSAIDs act mainly by inhibiting COX-2 and reducing PGE_2_ synthesis.

In contrast, HP has been reported to exhibit anti-inflammatory and analgesic activity through multiple molecular pathways in preclinical studies. Although HP appear to exert anti-inflammatory effects partly through modulation of COX-related pathways, experimental evidence suggests that HP constituents, including iridoid glycosides such as harpagoside, may additionally influence a broader range of inflammatory mediators. These include lipoxygenase-mediated eicosanoid production, pro-inflammatory cytokine expression (e.g., TNF-α, IL-1β, and IL-6), and transcriptional regulators associated with immune and inflammatory responses, such as NF-κB and AP-1 (Quarta et al., 2022; Fiebich et al., 2012; Haseeb et al., 2017; Inaba et al., 2010; Kaszkin et al., 2004; Schopohl et al., 2016; Rahimi et al., 2016; Loew et al., 2001). Several *in vitro* and animal studies have also suggested potential modulation of signaling pathways related to immune regulation, including PPAR-γ and CB2 receptor activation (Kim and Park, 2015; Mariano et al., 2022; Mariano et al., 2020). Collectively, these findings indicate that HP may interact with multiple inflammatory signaling pathways beyond prostaglandin inhibition (Figure 1).

Most studies investigating HP have employed heterogeneous experimental models, non-standardized extracts, and variable dosing regimens, limiting direct comparison across studies. In addition, some experimental studies have suggested that metabolic transformation of HP constituents, including conversion of harpagoside into active metabolites such as harpagogenin, may influence pharmacological activity; however, the pharmacokinetic and clinical relevance of these observations remains insufficiently characterized (Loew et al., 2001). Furthermore, several reports have indicated that whole-plant extracts may exert synergistic effects that are not reproducible with isolated compounds alone, further complicating mechanistic interpretation and standardization of HP preparations (Bargsten and Seifert, 2025; Alrumaihi et al., 2024).

Importantly, the clinical significance of these multi-target mechanisms remains uncertain. Although one randomized clinical trial reported that a standardized HP extract demonstrated efficacy comparable to that of diacerein with fewer reported adverse events in patients with osteoarthritis (Chantre et al., 2000), the overall comparative evidence base remains limited and methodologically heterogeneous. Therefore, current evidence remains insufficient to clearly define the comparative therapeutic role of HP relative to established NSAID therapies.

### 3.2 Therapeutic efficacy vs. NSAIDs

Among the 20 clinical studies identified to date, only a limited number directly compared HP monotherapy with established anti-inflammatory therapies, including NSAIDs and diacerein. Most available studies evaluated HP either as a monotherapy or as part of combination formulations, frequently using placebo controls or lacking active comparators (Supplementary Table S7). Because the therapeutic effects observed in combination-product studies cannot be confidently attributed specifically to HP owing to the concomitant use of multiple bioactive ingredients, studies evaluating HP monotherapy and HP-containing combination formulations were interpreted separately throughout the present review to minimize attribution bias.

#### 3.2.1 Evidence from HP monotherapy studies

Direct comparative studies of HP monotherapy have primarily involved patients with osteoarthritis or chronic low back pain (Table 1). In a 6-week randomized trial involving 88 patients with chronic low back pain, a standardized HP extract (Doloteffin®) demonstrated improvements in pain-related outcomes and functional indices that were broadly comparable to those observed with rofecoxib treatment (Chrubasik et al., 2003). Another 16-week randomized study involving 122 patients with osteoarthritis compared HP (Harpadol®) with diacerein and reported similar improvements in pain and functional outcomes between treatment groups, while NSAID rescue medication use and reported adverse events were lower in the HP group (Chantre et al., 2000). Similarly, a 4-week randomized controlled trial involving 60 patients with knee osteoarthritis found that HP extract (Teltonal®) and meloxicam produced improvements of similar magnitude in pain and WOMAC scores (Ouitas and Heard, 2010).

**TABLE 1:** HP monotherapy comparative studies.

HP intervention	Comparator	Study design	Sample size (n)	Follow-up duration	Main efficacy findings	Comparative interpretation	References
Harpadol® 2,610 mg/day (harpagoside 57 mg)	Diacerein 100 mg/day	RCT	122	16 weeks	Both groups demonstrated reductions in pain and Lequesne Index scores; lower rescue diclofenac use observed in the HP group	Symptomatic improvement appeared comparable under study conditions; long-term comparative efficacy remains uncertain	Chantre et al. (2000)
Doloteffin® 2,400 mg/day (harpagoside 60 mg)	Rofecoxib 12.5 mg/day	RCT, DB	88	6 weeks	Pain reduction and HAQ improvement reported in both groups; higher proportion of pain-free patients in the HP group	Some outcomes favored HP; however, direct equivalence with NSAIDs cannot be established because of limited sample size and study duration	Chrubasik et al. (2003)
Teltonal® 960 mg/day	Meloxicam 15 mg/day	RCT, AC	60	8 weeks	Similar reductions in VAS and WOMAC scores were observed between groups	No significant between-group difference was reported; interpretation limited by small sample size	Farpour et al. (2021)
HP extract 6,000 mg/day (harpagoside 50 mg)	Placebo	RCT, DB, PC	109	4 weeks	Increased proportion of pain-free patients and improvement in Arhus Index compared with placebo	Findings suggest short-term symptomatic benefit versus placebo	Chrubasik et al. (1996)

Abbreviations: VAS, visual analogue scale; WOMAC, Western ontario and mcMaster universities osteoarthritis index; RCT, randomized controlled trial; DB, Double-blind; AC, Active-controlled; PC, Placebo-controlled; HP, harpagophytum procumbens; MCID, minimal clinically important difference.

Placebo-controlled studies evaluating HP monotherapy have also been conducted in osteoarthritis, chronic low back pain, and rheumatic disorders (Chrubasik et al., 1996; Laudahn and Walper, 2001; Warnock et al., 2007; Wegener and Lüpke, 2003). In a 12-week randomized study involving 75 patients with hip and knee osteoarthritis, treatment with a standardized HP extract (Doloteffin®) was associated with improvements in pain and functional outcomes compared with placebo (Wegener and Lüpke, 2003). Additional placebo-controlled studies in chronic non-radicular and acute exacerbations of low back pain similarly reported reductions in pain-related outcomes following HP treatment (Chrubasik et al., 1996; Laudahn and Walper, 2001). In patients with rheumatic disorders, HP administration was additionally associated with reduced use of conventional analgesic medications during follow-up (Warnock et al., 2007). Although these findings suggest potential symptomatic benefit, most studies involved relatively small sample sizes and short follow-up durations, and the magnitude of benefit varied across clinical settings.

#### 3.2.2 Evidence from combination-product studies

Several combination formulations containing HP together with other botanical compounds have additionally been investigated in musculoskeletal disorders (Table 2) (Bender et al., 2024; González-Gross et al., 2021; Moré et al., 2017; Puigdellívol Grifell et al., 2024; Żęgota et al., 2021). Some of these studies reported improvements in pain-related outcomes, reductions in rescue medication use, or functional improvement. However, interpretation of these findings remains substantially limited because the observed therapeutic effects cannot be specifically attributed to HP owing to the concomitant use of multiple bioactive ingredients and adjunctive interventions. Additional topical formulations containing HP have also been investigated, although the available evidence remains limited and methodologically heterogeneous. Moreover, important variability exists across studies in formulation type, extraction methods, total extract dose, harpagoside concentration, comparator selection, study duration, and outcome measures, making direct comparison between studies challenging. Consequently, although several randomized studies reported symptomatic improvements under specific study conditions, the current evidence remains insufficient to establish standardized HP preparations as therapeutic alternatives equivalent to NSAIDs, and further large-scale comparative studies using well-characterized formulations remain necessary.

**TABLE 2:** HP-containing combination-product studies.

HP intervention	Comparator	Study design	Sample size (n)	Follow-up duration	Main efficacy findings	Comparative interpretation	References
MA212 (Rosaxan®) containing HP extract 108 mg	Placebo	RCT, DB, PC	90	6–12 weeks	Greater WOMAC improvement and reduced analgesic usage days compared with placebo	Suggests symptomatic improvement in osteoarthritis-related outcomes	Moré et al. (2017)
Sport cream containing HP	Standard management or diclofenac patch	RCT	95	2 weeks	Reduced emergency diclofenac usage observed in HP-treated patients	Potential reduction in rescue NSAID use reported under study conditions	Hu et al. (2021)
Loxacon® containing HP extract 270 mg	Physiotherapy ± placebo	RCT, DB, PC	88	5 weeks + follow-up	Greater MCID achievement reported in the HP combination group	Suggests adjunctive symptomatic benefit when combined with physiotherapy	Bender et al. (2024)

Abbreviations: VAS, visual analogue scale; WOMAC, Western ontario and mcMaster universities osteoarthritis index; RCT, randomized controlled trial; DB, Double-blind; AC, Active-controlled; PC, Placebo-controlled; HP, harpagophytum procumbens; MCID, minimal clinically important difference.

### 3.3 Safety profile vs. NSAIDs

Available clinical and preclinical evidence suggests that HP is generally well tolerated; however, the current evidence base has several important limitations that should be considered when interpreting its safety profile (Table 3). In a systematic review of 28 clinical trials involving 6,892 patients, the incidence of adverse events in HP-treated groups was reported to be comparable to that in placebo groups in double-blind studies, with approximately 3% of patients experiencing mainly mild adverse events (Vlachojannis et al., 2008). The most frequently reported adverse effects included mild gastrointestinal symptoms such as diarrhea, nausea, and abdominal discomfort (Mncwangi et al., 2012) Another systematic review further suggested that administration of HP extracts for up to 1 year was not associated with a higher incidence of adverse events compared with placebo (Cameron et al., 2009).

**TABLE 3:** Comparative safety and tolerability evidence of HP versus NSAIDs or conventional therapies.

Formulation category	HP intervention	Comparator	Study design	Sample size (n)	Main safety findings	Comparative interpretation	References
Monotherapy	Harpadol® 2,610 mg/day (harpagoside 57 mg)	Diacerein 100 mg/day	RCT	122	Lower overall AE rate and lower incidence of diarrhea observed in the HP group	HP demonstrated generally favorable gastrointestinal tolerability under study conditions	Chantre et al. (2000)
	Doloteffin® 2,400 mg/day (harpagoside 60 mg)	Rofecoxib 12.5 mg/day	RCT, DB	88	Similar overall AE rates reported between groups; fewer discontinuations observed in the HP group	Comparable short-term tolerability was reported; interpretation limited by small sample size	Chrubasik et al. (2003)
	HP extract 6,000 mg/day (harpagoside 50 mg)	Placebo	RCT, DB, PC	109	Lower total AE events observed in the HP group compared with placebo	Findings suggest acceptable short-term tolerability under study conditions	Chrubasik et al. (1996)
Combination	MA212 (Rosaxan®) containing HP extract 108 mg	Placebo	RCT, DB, PC	90	Lower AE incidence and no treatment discontinuation reported in the HP group	Suggests generally favorable tolerability profile	Moré et al. (2017)
	Artipotect® containing HP extract 150 mg	None	Single-arm, OL	130	Low incidence of gastrointestinal adverse events and high medication adherence reported	Long-term tolerability appeared acceptable in this observational study	Puigdellivol et al. (2019)
	Tregocel® containing HP extract 1,000 mg	None	Single-arm, OL	137	Gastrointestinal and laboratory-related adverse events were reported at relatively low frequency	Observational findings suggest generally acceptable tolerability	Żęgota et al. (2021)
	Artipotect Forte® containing HP extract 150 mg	None	Single-arm, OL	186	Very low AE incidence and high medication adherence reported	Suggests favorable patient-reported tolerability	Puigdellívol Grifell et al. (2024)

Abbreviations: VAS = RCT, randomized controlled trial; DB, Double-blind; AC, Active-controlled; PC, Placebo-controlled; AEs, Adverse events; HP, harpagophytum procumbens; OL, Open-label.

Several comparative clinical studies evaluating standardized HP extract monotherapy have suggested generally acceptable short-term tolerability relative to conventional therapies; however, the available comparative evidence remains limited and heterogeneous. In one study, both the Doloteffin™ and rofecoxib groups reported nine cases of gastrointestinal symptoms, although treatment discontinuation occurred more frequently in the rofecoxib group (Chrubasik et al., 2003). Another comparative trial reported diarrhea in 8.1% of HP users compared with 26.7% in the diacerein group, with a lower treatment discontinuation rate due to adverse effects in the HP group (Chantre et al., 2000). Similarly, placebo-controlled studies evaluating standardized HP extracts reported relatively low incidences of mild gastrointestinal adverse events under study conditions (Vlachojannis et al., 2008; Mncwangi et al., 2012; Cameron et al., 2009). Nevertheless, these findings should be interpreted cautiously because the number of direct comparative trials remains limited, and differences in study design, patient populations, treatment duration, and extract standardization reduce the generalizability of the results.

Several additional studies evaluated HP-containing combination formulations, including Rosaxan®, Artipotect®, Artipotect Forte®, and Tregocel® (Moré et al., 2017; Puigdellívol Grifell et al., 2024; Żęgota et al., 2021; Puigdellivol et al., 2019). These studies generally reported low incidences of adverse events and favorable patient-reported tolerability during follow-up. However, because these formulations contained additional bioactive compounds or adjunctive interventions, the reported safety outcomes cannot be specifically attributed to HP alone. Accordingly, these studies were interpreted separately and were not considered primary evidence supporting comparative safety conclusions between HP and NSAIDs.

Importantly, rare adverse events associated with HP have also been reported. Cases of gastrointestinal phytobezoar-induced bowel obstruction following long-term use have been described (Bender et al., 2024), and one case report documented stage 2 hypertension in a 62-year-old woman after 2 weeks of HP use (Wegener and Lüpke, 2003). In addition, high-dose use during pregnancy is not recommended because of insufficient safety data (González-Gross et al., 2021).

Preclinical toxicological studies have also suggested relatively low acute and subchronic toxicity of HP extracts. The median lethal dose (LD_50_) in mice was reported to exceed 13.5 g/kg, indicating low acute toxicity (Chrubasik et al., 1996). In repeated-dose toxicity studies conducted over 1- and 3-month periods, no significant pathological changes were observed in the liver, kidneys, or gastrointestinal tract (Laudahn and Walper, 2001). Nevertheless, interpretation of these findings requires caution because many toxicological studies used heterogeneous extract formulations and experimental conditions. Furthermore, significant sex-related differences in blood chemistry were observed in one study, with increased potassium levels in males and elevated phosphate and bilirubin levels in females, suggesting the need for further investigation regarding sex-specific safety considerations (Laudahn and Walper, 2001).

By contrast, NSAIDs are associated with well-established gastrointestinal, cardiovascular, and renal toxicities. Gastrointestinal adverse events have been reported in approximately 20%–40% of NSAID users (Langmead et al., 2002), and severe complications such as gastrointestinal bleeding, ulceration, and perforation may occur. NSAID-related gastrointestinal toxicity has been estimated to contribute to approximately 12,000 hospitalizations and 2,000 deaths annually in the UK (Warnock et al., 2007). Furthermore, selective COX-2 inhibitors have been associated with increased risks of cardiovascular events and hypertension (Wegener and Lüpke, 2003).

Overall, currently available evidence suggests that standardized HP preparations may exhibit acceptable short-term tolerability under selected study conditions. However, the comparative safety evidence remains limited by heterogeneous formulations, variable study designs, and inclusion of combination-product studies, highlighting the need for larger well-controlled studies using standardized preparations and rigorous long-term safety assessment.

### 3.4 Pharmacokinetics and metabolism vs. NSAIDs

The pharmacokinetic and metabolic characteristics of HP have been investigated in a limited number of preclinical and clinical studies. Although several pharmacokinetic features appear to differ from those of conventional NSAIDs, direct comparisons remain challenging because of variability in HP preparations, study designs, analytical methodologies, and the limited availability of standardized human pharmacokinetic data.

Harpagoside, one of the principal iridoid glycosides in HP, reportedly reaches maximum plasma concentrations (Cmax) within approximately 1.3–2.5 h after oral administration, with an elimination half-life (t_1_/_2_) of 4–6 h (Loew et al., 2001). However, its oral bioavailability appears relatively low, and doses below 54 mg may not be readily detectable in systemic circulation, potentially owing to poor intestinal absorption or first-pass metabolism. These findings have led to suggestions that repeated daily administration may be required to maintain therapeutic exposure, although the relationship between plasma concentrations and clinical efficacy remains insufficiently characterized.

A substantial proportion of the currently available pharmacokinetic evidence for HP originates from animal studies, and the translational applicability of these findings to humans remains uncertain. In a preclinical study in horses, harpagoside was detected in plasma within 30 min after oral administration and demonstrated dose-dependent pharmacokinetic behavior. At doses of 5 and 10 mg/kg, the reported Cmax values were 25.59 and 55.46 ng/mL, respectively, whereas the volume of distribution (Vd) ranged from 259 to 284 L/kg (Axmann et al., 2019). These findings may suggest extensive tissue distribution; however, their clinical relevance in humans remains unclear. By comparison, many NSAIDs are characterized by relatively rapid gastrointestinal absorption and typically reach peak plasma concentrations within 1–2 h (Wongrakpanich et al., 2018). For example, ibuprofen exhibits a Tmax of approximately 2 h following oral administration (Rainsford, 2009). NSAIDs are also generally associated with high plasma protein binding, particularly to albumin, which contributes to relatively limited distribution volumes (Calatayud and Esplugues, 2016).

The metabolic pathways of HP constituents also remain incompletely understood. Harpagoside is believed to undergo hydrolysis to harpagogenin, which has been proposed as a potentially active metabolite contributing to anti-inflammatory activity through esterase-mediated processes (Loew et al., 2001). In contrast, many NSAIDs undergo hepatic metabolism primarily via cytochrome P450 (CYP)-mediated oxidation and conjugation reactions, producing metabolites that are subsequently eliminated through renal or biliary excretion. Certain NSAIDs, including sulindac and parecoxib, also require hepatic activation to exert pharmacological activity (Brune and Patrignani, 2015). However, the clinical significance of these mechanistic differences between HP and NSAIDs has not yet been clearly established.

Stability studies have shown that harpagoside undergoes partial degradation in artificial gastric fluid but remains relatively stable in artificial intestinal fluid (Chrubasik et al., 2000), suggesting that intestinal absorption may contribute substantially to systemic exposure. In addition, several *in vitro* studies have reported that HP extracts may inhibit multiple CYP isoforms, including mild inhibition of CYP1A2 and CYP2D6 and moderate inhibition of CYP2C8, CYP2C9, CYP2C19, and CYP3A4 (Sato et al., 2015; Unger and Frank, 2004). HP has also been reported to inhibit P-glycoprotein (P-gp), potentially affecting the transport of co-administered compounds (Romiti et al., 2009). Although currently available studies suggest that clinically significant herb–drug interactions may be uncommon, the available evidence remains limited, and caution may still be warranted in patients receiving medications metabolized through CYP pathways or transported by P-gp.

NSAIDs are also associated with clinically important pharmacokinetic interactions, largely because of their high protein-binding affinity and hepatic metabolism. For example, aspirin may alter the bioavailability or pharmacodynamic effects of co-administered NSAIDs, and interactions with anticoagulants and oral hypoglycemic agents have been documented (Brune and Patrignani, 2015). Furthermore, genetic polymorphisms, particularly involving CYP2C9, have emerged as important determinants of interindividual variability in NSAID metabolism and toxicity risk (Agúndez et al., 2021).

Current evidence suggests that HP possesses pharmacokinetic characteristics distinct from those of conventional NSAIDs, including relatively low oral bioavailability, potential extensive tissue distribution in animal studies, and modulation of CYP enzymes and drug transporters. However, interpretation of these findings remains limited by the small number of human pharmacokinetic studies, variability among commercial preparations, and insufficient standardization across studies. Further well-designed pharmacokinetic and pharmacodynamic investigations are necessary to clarify the clinical implications of these findings and their relevance to efficacy, safety, and herb–drug interactions.

### 3.5 Regulatory and clinical use vs. NSAIDs

HP and NSAIDs are regulated under substantially different approval frameworks, reflecting differences in historical use, evidence requirements, product classification, and post-marketing surveillance systems. In Europe, the Committee on Herbal Medicinal Products (HMPC) of the European Medicines Agency (EMA) has concluded that HP root preparations may be used for the relief of mild joint pain based primarily on long-standing traditional use (Bargsten and Seifert, 2025). According to the HMPC monograph, HP preparations have been used for at least 30 years, including a minimum of 15 years within the European Union, without the requirement for direct medical supervision in adults. In addition, organizations such as Commission E and the European Scientific Cooperative on Phytotherapy (ESCOP) have recommended HP for conditions including osteoarthritis, rheumatism, and musculoskeletal pain (Wegener and Lüpke, 2003; Axmann et al., 2019). In Germany, certain HP products are available as over-the-counter (OTC) herbal medicinal products for degenerative musculoskeletal disorders (Quarta et al., 2022; Lubecka and Szmeja, 2018).

Importantly, traditional-use registration under the HMPC framework does not necessarily require the same level of clinical efficacy evidence as that required for conventional pharmaceutical approval. Instead, approval is largely based on longstanding use, acceptable safety profiles, and plausibility of therapeutic effects. Under the European Pharmacopoeia, HP preparations are required to contain at least 1.2% harpagoside (Joshi et al., 2020), and the EMA recognizes several dosage forms, including powdered root materials and ethanol or aqueous extracts intended for oral administration (Avato and Argentieri, 2019). However, the regulatory classification of HP products varies considerably across jurisdictions, where they may be marketed as herbal medicinal products (HMPs), food supplements (FS), or dietary supplements (DS). Consequently, manufacturing standards, labeling requirements, premarketing evaluation procedures, and post-marketing surveillance systems may differ substantially between countries.

By contrast, NSAIDs are regulated as conventional pharmaceutical agents by authorities such as the EMA and the U.S. Food and Drug Administration (FDA), with approval based on extensive preclinical and clinical efficacy and safety data (Ghlichloo and Gerriets, 2025). NSAIDs are indicated for a broad spectrum of inflammatory and pain-related conditions, including osteoarthritis, dysmenorrhea, gout, migraine, posttraumatic pain, and musculoskeletal disorders. Their clinical use is also guided by extensive pharmacovigilance systems and regularly updated safety warnings. Accumulating evidence has associated NSAIDs with gastrointestinal, renal, hepatic, and cardiovascular adverse effects, including increased cardiovascular risk following myocardial infarction (Olry de Labry Lima et al., 2021; Kang et al., 2020). These stringent safety oversight mechanisms contrast with the relatively simplified regulatory systems governing HP.

Clinically, HP is administered in various formulations, including tablets, capsules, powders, and extracts, with commonly used preparations providing approximately 50–60 mg/day of harpagoside (Chantre et al., 2000; Chrubasik et al., 2003; Chrubasik et al., 1996; Wegener and Lüpke, 2003). However, substantial variability exists among commercial products with respect to extract composition, dosage standardization, and labeling practices. In some regions, particularly where HP products are marketed as dietary supplements rather than registered herbal medicines, premarketing efficacy evaluation and standardized contraindication labeling may be limited. Although widespread OTC availability may improve accessibility, it may also contribute to inconsistencies in product quality, safety assessment, and manufacturing oversight across markets (Wilson, 2009).

Certain precautions regarding HP use have also been reported. HP is generally not recommended during pregnancy and is often contraindicated in children and individuals with gastrointestinal disorders (Verhaar et al., 2021). Nevertheless, contraindications and safety information may not be uniformly presented across commercially available products. These differences in regulatory classification and quality control standards may partially explain the variability in available clinical evidence and post-marketing safety data for HP compared with NSAIDs.

HP occupies a distinct regulatory and clinical position from NSAIDs, reflecting differences in historical use, evidence requirements, and product classification systems. While HP is widely used for mild musculoskeletal symptoms in several countries, important challenges remain regarding product standardization, consistency of clinical evidence, and long-term safety evaluation.

### 3.6 Cost-effectiveness and patient preference vs. NSAIDs

The potential cost-effectiveness of HP relative to NSAIDs has attracted increasing interest, particularly in the context of long-term management of musculoskeletal disorders. However, the currently available economic evidence remains limited because relatively few studies have directly evaluated the comparative cost-effectiveness of HP and conventional NSAIDs using standardized pharmacoeconomic methodologies.

The market price of HP products varies considerably depending on manufacturing standards, extraction methods, certification status, and regulatory classification. Reported costs per kilogram range from approximately €6.70 for standard-grade material to €9.00 for Fair for Life-certified products (Brendler, 2021). Across dosage forms, average unit prices have been reported to range from €0.52 for food supplements to approximately €0.95 for herbal medicinal products, reflecting differences in production standards and regulatory oversight (Bargsten and Seifert, 2025). However, direct comparisons of medication costs between HP and NSAIDs remain difficult because commercial products differ substantially in formulation, harpagoside content, dosing regimens, and quality control standards.

In contrast, the economic burden associated with NSAID therapy extends beyond the direct cost of medication and includes expenditures related to the prevention and management of adverse events, particularly gastrointestinal, cardiovascular, and renal complications (Herings and Klungel, 2001). In patients at increased gastrointestinal risk, co-prescription of gastroprotective agents such as proton pump inhibitors (PPIs) or misoprostol is often recommended, potentially increasing overall treatment costs (Scheiman, 2013). Additional monitoring for renal, hepatic, or cardiovascular complications may further contribute to healthcare expenditures during long-term NSAID therapy. Nevertheless, the magnitude of these costs varies substantially depending on patient risk profiles, duration of treatment, and healthcare system structure.

In a 6-week comparative study evaluating a standardized HP extract (Doloteffin®) and rofecoxib, overall treatment costs were reported to be broadly similar between groups, although the HP group required fewer additional interventions related to adverse effects under the study conditions (Chrubasik et al., 2003).

Several studies evaluating HP-containing combination formulations have reported favorable patient acceptance and adherence outcomes. In one clinical trial involving the MA212 preparation, 85% of participants reported “very good” adherence compared with 51% in the placebo group (Moré et al., 2017). Another study reported that 63.1% of patients consumed 75%–100% of the prescribed HP dosage, whereas 74.3% expressed willingness to reuse the product in the future (Warnock et al., 2007). However, because these studies involved combination formulations or observational designs, the observed adherence and patient preference outcomes cannot be specifically attributed to HP alone. In addition, direct comparisons with adherence rates for NSAIDs remain difficult because adherence is influenced by multiple clinical and behavioral factors, including symptom severity, treatment duration, treatment expectations, and patient perceptions regarding herbal therapies.

Some observational studies involving HP-containing combination formulations have additionally reported reductions in concomitant NSAID use during follow-up (Żęgota et al., 2021). However, because these studies included additional active compounds and lacked rigorous comparative controls, the observed reductions in NSAID consumption cannot be confidently attributed specifically to HP.

Patient preference for HP may also be influenced by perceptions regarding natural therapies, tolerability, and suitability for long-term use. For example, among medicinal plants used for gout management, HP ranked highly in physician preference surveys (Corp and Pendry, 2013). However, patient and physician preference data are inherently subjective and may vary across healthcare systems, cultural settings, and clinical contexts.

HP may represent a potentially useful complementary option for selected patients with mild musculoskeletal symptoms, particularly in situations where long-term NSAID therapy is poorly tolerated or contraindicated. Nevertheless, current evidence regarding the comparative cost-effectiveness and patient preference of HP remains limited and heterogeneous. In particular, several reported adherence and NSAID-reduction outcomes were derived from studies evaluating HP-containing combination formulations, limiting attribution specifically to HP. Additional well-designed pharmacoeconomic studies using standardized HP preparations and rigorous comparative methodologies are necessary to clarify the economic and clinical relevance of HP in musculoskeletal care.

## 4 Discussion

This review critically evaluated the currently available preclinical and clinical evidence regarding the pharmacological properties, clinical efficacy, safety, pharmacokinetics, regulatory considerations, and potential therapeutic applications of HP in musculoskeletal disorders, with particular emphasis on comparative evidence involving NSAIDs.

Preclinical investigations have suggested that HP may exert anti-inflammatory effects through modulation of multiple signaling pathways, including COX-2 inhibition, suppression of NF-κB and MAPK signaling, and regulation of inflammatory cytokines and oxidative stress pathways. In addition, analgesic activity has been observed in nociceptive, inflammatory, and neuropathic pain models, potentially involving opioid receptors, TRPV1, and the HO-1/CO system. Most currently available mechanistic evidence has been derived from *in vitro* or animal studies, and the translational relevance of these findings to human clinical outcomes remains incompletely established. Furthermore, the diverse phytochemical composition of HP suggests the possibility of multi-target pharmacological activity; however, the precise contribution of individual constituents and their potential synergistic interactions remain insufficiently characterized.

Several clinical studies have suggested that certain HP preparations may provide symptomatic improvement in conditions such as osteoarthritis, chronic low back pain, and inflammatory musculoskeletal disorders. Some comparative studies also reported clinical outcomes that appeared comparable to those observed with selected NSAIDs or symptomatic slow-acting drugs under specific study conditions. Nevertheless, the currently available clinical evidence remains insufficient to establish definitive comparative efficacy because of the limited number of standardized head-to-head trials.

Currently available evidence generally suggests favorable tolerability of HP preparations, with most reported adverse events consisting of mild gastrointestinal symptoms. Nevertheless, the available safety evidence remains limited by relatively short-term follow-up periods, insufficient pharmacovigilance data, and limited evaluation of rare or delayed adverse events. In contrast, NSAIDs are supported by a substantially larger body of evidence documenting both efficacy and adverse event profiles, including gastrointestinal, cardiovascular, renal, and hepatic toxicities. Although some studies have suggested lower rates of gastrointestinal adverse effects with HP than with certain conventional therapies, these observations should be interpreted cautiously because of differences in study methodology, patient populations, and product standardization.

The pharmacokinetic characteristics of HP also remain incompletely understood. Available evidence suggests relatively low oral bioavailability of harpagoside, possible extensive tissue distribution in animal studies, and potential modulation of CYP enzymes and drug transporters. However, most pharmacokinetic studies have been preclinical, and direct extrapolation to clinical settings remains uncertain. Similarly, although current evidence suggests that clinically significant herb–drug interactions may be uncommon, additional investigation is required, particularly in patients receiving multiple concomitant medications.

From a regulatory perspective, HP occupies a distinct position from conventional NSAIDs. In several countries, HP is recognized as a traditional herbal medicinal product based primarily on longstanding historical use and acceptable safety data. Importantly, traditional-use registration does not necessarily require the same degree of clinical efficacy evidence as that required for conventional pharmaceutical approval. In addition, considerable variability exists across commercial HP products regarding extract composition, harpagoside content, manufacturing standards, and regulatory oversight. These differences may contribute to inconsistencies in clinical outcomes and complicate cross-study comparisons.

Economic and patient preference data regarding HP also remain limited. Some studies have suggested favorable patient adherence and the potential for reduced healthcare utilization associated with adverse event management. Additional pharmacoeconomic investigations and long-term comparative studies will be important to better define the economic and patient-centered value of HP in musculoskeletal care.

Several important limitations in the current evidence base should be acknowledged. First, many studies evaluating HP involve heterogeneous formulations, extraction methods, and dosing regimens, limiting reproducibility and comparability across investigations. Second, relatively few large-scale, high-quality randomized controlled trials directly comparing HP with NSAIDs have been conducted. Third, many available studies focus primarily on chronic musculoskeletal conditions, whereas evidence regarding acute inflammatory disorders, severe disease states, or central pain syndromes remains insufficient. In addition, long-term safety data and standardized pharmacovigilance systems for HP are currently limited.

Future research should therefore focus on several key priorities. Mechanistic studies using systems biology, transcriptomics, and metabolomics approaches are needed to better characterize the molecular targets and synergistic interactions of HP constituents. In parallel, the development of standardized formulations with consistent phytochemical composition and pharmacokinetic profiles is important for improving reproducibility and quality control. Clinically, large-scale and rigorously designed randomized controlled trials comparing HP with conventional and selective NSAIDs across diverse patient populations are required. Additional studies evaluating long-term safety, comparative effectiveness, pharmacoeconomic outcomes, and potential combination strategies with conventional analgesics should also be prioritized. Furthermore, harmonization of regulatory standards and strengthened post-marketing surveillance systems may help improve consistency in product quality and clinical evidence generation.

Overall, HP may represent a potentially useful complementary therapeutic option for selected patients with mild to moderate musculoskeletal symptoms, particularly in situations where long-term NSAID therapy is poorly tolerated or contraindicated. Although additional standardized comparative studies are still needed, currently available evidence supports continued investigation of HP as part of an integrative therapeutic approach for chronic musculoskeletal disorders.
