# Correction: Harpagophytum procumbens in musculoskeletal disorders: current evidence and comparison with NSAIDs

**DOI:** 10.3389/fphar.2026.1930236

**Published:** 2026-07-28

**Authors:** Jin Young Hong, Junseon Lee, Hyunseong Kim, Hyun Kim, Wan-Jin Jeon, Changhwan Yeo, Yoon Jae Lee, Ho-Yeon Go, In-Hyuk Ha

**Affiliations:** 1 Jaseng Spine and Joint Research Institute, Jaseng Medical Foundation, Seoul, Republic of Korea; 2 Department of Korean Internal Medicine, College of Korean Medicine, Semyung University, Jecheon-si, Republic of Korea

**Keywords:** alternative, devil’s claw, harpagophytum procumbens, inflammation, NSAIDs

There was a mistake in Scheme 1 and Figure 1 as published. The images for Scheme 1 and Figure 1 were inadvertently interchanged during the publication process; the image displayed as Scheme 1 should be Figure 1, and the image displayed as Figure 1 should be Scheme 1.

**SCHEME 1:**
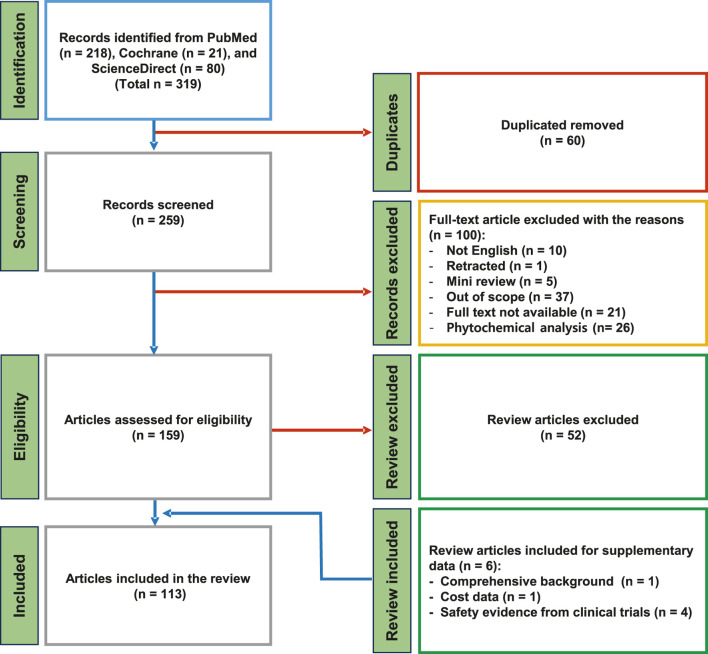
Overview of the literature search, screening, categorization, and narrative synthesis process used in this structured narrative review of Harpagophytum procumbens and NSAIDs.

**FIGURE 1:**
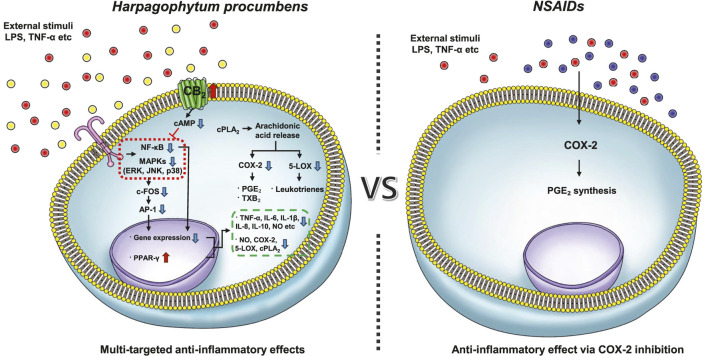
Schematic representation of the anti-inflammatory mechanisms of *HP* compared to NSAIDs. HP exerts anti-inflammatory effects through multiple pathways, including inhibition of MAPKs and NF-κB signaling, activation of PPAR-γ, and suppression of pro-inflammatory mediators (e.g., cytokines, COX-2, 5-LOX, cPLA2). In contrast, NSAIDs act mainly by inhibiting COX-2 and reducing PGE2 synthesis.

The corrected Scheme 1 and Figure 1 appear below.

The original version of this article has been updated.

