# Supplementary material for: Harpagophytum procumbens in musculoskeletal disorders: current evidence and comparison with NSAIDs
Source: Front Pharmacol. 2026 Jun 17;17:1839470. doi: 10.3389/fphar.2026.1839470 (PMC13323821; doi:10.3389/fphar.2026.1839470)
Supplement: Supplementary file 1 [file Table1.docx]

**Table S1. Anti-inflammatory activity of HP vs. NSAIDs: In vitro & ex vivo evidence**

| Study Type | NSAID Comparison | Key comparator | Cell/Tissue Type | Injury Inducer | HP Treatment | Biomarkers | Key Findings Summary | Reference |
| --- | --- | --- | --- | --- | --- | --- | --- | --- |
| In vitro | Yes | Indomethacin (40 μM), Etoricoxib (300 μM) | Human whole blood | LPS, 100 µg/mL | 60% ethanol: 0.625-10 µg/mL Fractions A: 30, 100, 300 µg/mL Fractions B: 30, 100, 300 µg/mL Fractions C: 30, 100, 300 µg/mL | COX-1, COX-2, TXB2, PGE2 | Crude extract: no COX-1/2 inhibition  Fraction A: ↓ COX-1/2 (30–37%)  Fraction B/C: no COX-1/2 effect  HP extracts: less COX inhibition vs. indomethacin/etoricoxib | Anauate et al., 2010 (1) |
|  | NO | - | Primary human monocytes | LPS, 10 ng/ml | PSH69 (purified harpagophytum extract): 10–1000 µg/mL, Harpagoside, harpagide: 0.01-10 µg/mL | IL-6, IL-1β, PGE₂, TNF-α | PSH69: ↓ TNF-α (IC₅₀ ≈ 100 μg/mL), ↓ IL-6/IL-1β/PGE₂ (≥100 μg/mL)  Harpagoside / Harpagide : no effect on TNF-α (10 μg/mL) | Fiebich et al., 2001 (2) |
|  | NO | - | L929 | LPS, 5 μg/mL | Water ectract: 0.1, 1 mg/mL | COX-1, COX-2, PGE2 | HP (1 mg/mL): ↓ COX-1 mRNA (0.67-fold)  HP: ↓ COX-2 mRNA (11.96 → 0.95 vs. LPS)  HP: ↓ PGE₂ (71.00 → 22.00 pg/well vs. LPS) | Jang et al., 2003 (3) |
|  | NO | - | Rat mesangial cell | IL-1β, 0.5 nM | Extract1-5 | NF-κB | Extracts 1, 2, & harpagoside-free: ↓ NF-κB activation (dose-dependent)  Extracts 3–5 & harpagoside alone: ↔ NF-κB (no effect) | Kaszkin et al., 2004 (4) |
|  | NO | - | MCF10A | TPA,10 nM | Methanolic extract (DEV): 1–10 µg/mL | COX-2 | DEV extract : ↓ TPA-induced COX-2 expression (MCF10A cells) | Na et al., 2004 (5) |
|  | NO | - | Human whole blood | - | Conventional extract (2.3% harpagoside), supercritical extract (29.7% harpagoside), subcritical extract (20.1% harpagoside): 0.0518-51.8 mg/L | PGE2 (Cox2 inhibition), LTB4 (5-LO inhibition) | PGE2 (Cox2 inhibition) - Conventional extract: ↓ Weak & inconsistent inhibition -Supercritical/  Subcritical extractzz; ↓ Weak inhibition  LTB4 (5-LO Inhibition), - Conventional extract: No inhibition - Supercritical and subcritical extract: Complete inhibition at 51.8 mg/L | Günther et al., 2006 (6) |
|  | NO | - | RAW 264.7, HepG2 | LPS, 100 ng/mL | Harpagoside: 0.1–200 μM | COX-2, NF-κB | - Harpagoside (200 µM)  : ↓ COX-2 mRNA & protein (HepG2)  : ↓ NF-κB activity (IC₅₀ ≈ 96.4 µM, RAW 264.7)  : ↑ IκB-α (cytosolic restoration) | Huang et al., 2006 (7) |
|  | NO | - | RAW 264.7 | LPS, 1 μg/mL | Ethanol extract (HP-ext): 50, 500, 500 µg/mL Harpagoside: 50, 100, 200 µM | IL-1β, IL-6, TNF-α | - HP-ext, harpagoside  : ↓ IL-1β, IL-6, TNF-α (LPS-stimulated RAW264.7)  : No cytotoxicity | Inaba et al., 2010 (8) |
|  | NO | - | Peritoneal macrophages | LPS, 1 μg/mL | 1. CME-CB  2. PF-CB  3. CME-HR  4. PF-HR 5.Verbascoside (Vs) 6.b-OH-verbascoside (b-OH-Vs) 7.Leucosceptoside A (Leuco)  8.Martynoside (Marty)  9.Harpagide; (Hr) 10. Harpagoside (Hs) |  | - NO ↓ all extracts & pure compounds (except CME-HR); PF-HR: strongest (↑8-fold vs. CME-HR)  - Phenylethanoids > Harpagosides, Leucopyranosides in NO inhibition  - TNF-α ↓: CME-CB, PF-CB, CME-HR, Vs, Marty  - IL-6 ↓: CME-CB, PF-CB > PF-HR; Hs no effect  - COX-1/2 ↓: all extracts & pure compounds; COX-2 inhibition comparable to Hs, Vs → biomass synergy | Valeriya Gyurkovska et al., 2011 (9) |
|  | NO | - | Human monocytes, RAW 264.7 | LPS, 10 ng/ml or 2 μg/mL | Ethanol extract: 10, 50, 100, 250, 500 µg/mL | IL-1β, IL-6, TNF-α, PGE2, NF-κB, MAPK, AP-1 | - TNF-α, IL-1β, IL-6, PGE₂ ↓ (IC₅₀ ≈ 100 µg/mL)  - COX-2 transcription ↓, AP-1 ↓ (high dose)  - NF-κB, MAPK no effect | Fiebich et al., 2012 (10) |
|  | NO | Dexamethasone (10-7M) | THP-1 | LPS, 25 μg/mL | Ethanl extract (DC), Metabolic extract (DCm): 10, 50, 100, 250 µg/mL | IL-1α, IL-1β, IL-2, IL-4, IL-6, IL-8, IL-10, IL-12, IL17A, IFN-γ, TNF-α | - DC, DCm: TNF-α, IL-6, IL-8 ↓ (dose-dependent)  - DCm > DC for TNF-α (EC₅₀: 49 vs. 116 µg/mL)  - IL-6, IL-8 ↓ only at 250 µg/mL (DC ≈ DCm). | Hostanska et al., 2014 (11) |
|  | NO | - | 3T3-L1 adipocytes | TNF-α, 10 ng/mL | Harpagoside: 5, 10, 20, 50 µM | IL-6, PAI-1, MCP-1 | - Harpagoside (20/50 µM): ↓ IL-6, MCP-1, PAI-1 (mRNA & ELISA)  - Effect reversed by GW9662 → PPAR-γ pathway involvement | Kim & Park, 2015 (12) |
|  | NO | - | THP-1 | IFNγ (10 ng/mL) + LPS (100 ng/mL) | Harpagoside, Harpagide: 5, 50, 500 µM | TNF-α, CD86, ICAM-1, PECAM-1, L-SEL, PSGL-1 | - Harpagoside: ↑ TNF-α, ICAM-1, L-selectin, PSGL-1 (THP-1, IFN-γ/LPS; sustained 13–17-fold ↑)  - Harpagide: similar but weaker effects | Schopohl et al., 2016 (13) |
|  | NO | - | Human chondrocytes | IL-1β, 0.1 or 10 ng/mL | Harpagoside: 300 µg/mL | IL-6, c-FOS/AP-1, MMP13 | - Harpagoside: ↓ IL-6, MMP-13 (IL-1β-induced)  - inhibited c-FOS/AP-1 only (no effect NF-κB, C/EBPβ) | Haseeb et al., 2017 (14) |
|  | NO | - | BV-2 | LPS, 100 ng/mL | Water extract: 10, 20, 40, 80 µg/mL | p-cPLA2/cPLA2 | - HP extract → ↓ p-cPLA₂ (LPS-stimulated BV-2) → inhibited arachidonic acid pathway | Ungerer et al., 2020 (15) |
|  | NO | - | Human primary synoviocytes | - | HPE_DMSO_  HPE_EXOH100_ HPE_EXOH50_ HPE_H2O_ | CB1, CB2, FAAH | - HPE_H₂O_, HPE_DMSO_: ↑ CB2, CB1 (weak); ↓ PI-PLCβ2; inhibited FAAH  - HPE_EtOH100/50_ → inhibited FAAH activity (significant) | Mariano et al., 2020 (16) |
|  | NO | - | Human keratinocytes | - |  | JAK2/STAT1, PI3K/AKT pathway | - Psoriasis-like model: ↑ JAK2, STAT1, PI3K; ↓ AKT  - HP, VER, LEU: ↓ total STAT1  - LEU → ↓ p-STAT1 (mild)  - HP (100 µg/mL), LEU (10 µg/mL): ↓ pro-inflammatory gene expression | Koycheva et al., 2021 (17) |
|  | NO | - | Human primary synoviocytes | Forskolin | HPE_DMSO_: 0.1 mg/mL Harpagoide: 0.4 µg/mL | cAMP, PKA, ERK, c-Fos, MMP13, CB2 | - HPE_DMSO_: ↓ cAMP, p-PKA (± forskolin); ↓ p-ERK, c-Fos  - Harpagoside: ↑ CB2; ↓ cAMP, MMP-13 mRNA | Mariano et al., 2022 (18) |
|  | NO | - | THP-1 | TNFα, 10 ng/mL | Flonat Fast® (HP, Boswellia, Curcuma, Escin, Bromelain; FF) Water extract (HP alone): 500 µg/mL | IL-6, IL-8, COX-2, MMP-9, VCAM-1, ICAM-1 | - HP, HP+FF: ↓ IL-6, IL-8, COX-2, MMP-9 (mRNA)  - HP: ↓ VCAM-1 (250 µg/mL > 500 µg/mL). | Quarta et al., 2022 (19) |
|  | NO | - | Human synoviocytes | Forskolin | HPE_DMSO_: 0.1 mg/mL | PI‐PLC‐γ, MMP3, MMP13, ADAMS-5 | - HPE_DMSO_: ↓ PI-PLCγ1, MMP-3, MMP-13, ADAMTS-5 (mRNA & protein) → Effect linked to intron retention in PI-PLCγ1. | Mariano et al., 2024 (20) |
| Ex vivo | YES | Ibuprofen (1 mg/ml) | Franz cell-based porcine skin | - | Ethanol extract: 1 mg/mL Harpagoside, harpagide. 8-coumaroylharpagide. Verbascoside,: 500μM | COX-2 | - Harpagoside, 8-Coumaroylharpagide: strongly reduced COX-2 expression (greater than verbascoside)  - Harpagide: significantly increased COX-2 expression (pro-inflammatory effect)  - All individual compounds (except harpagide): more effective than HP extract; comparable to ibuprofen | Abdelouahab & Heard, 2008 (21) |
|  | NO | - | Franz cell-based porcine skin | - | Ethanol extract: 1 mg/mL | COX-2, 5-LOX, PGE2 | - HP saturated extract: inhibited COX-2, PGE₂ (strong)  - Permeated glycosides (e.g., harpagide): ↓ COX-2 (moderate); no effect on 5-LOX, iNOS | Ouitas et al., 2009 (22) |
|  | NO | - | Porcine skin | - | Six different commercial extracts from UK-based vendors: Medic Herb (A), Boots (B), Viridian (C), Swiss Herbal Remedies (D), Good ‘N Natural (E), and Just Vitamins (F). | COX-2 | - Extracts D, F: ↑ COX-2 expression  - Extracts A–C, E (↑ harpagoside/verbascoside): ↓ COX-2 (trend, not significant). | Ouitas, N.A & Heard, C., 2010 (23) |
|  | NO | Sulfasalazine (2 mg/mL) | Rat colon tissue | LPS, 10 μg/mL | Microwave water extracts: 100, 500, 1000 µg/mL | PGE2, 5-HT, TNF-α | - HP extract (100–1000 μg/mL): inhibited LPS-induced PGE₂, 5-HT, TNF-α (colon tissue)  - Comparable efficacy to sulfasalazine (2 mg/mL); no clear dose-dependence | Locatelli et al., 2017 (24) |
|  | NO | Sulfasalazine (2μg/mL) | HCT116, Mouse colon tissue | LPS, 10 μg/mL | Water extract: 100, 500, 1000 µg/mL Harpagoside: 12 µg/mL | 5-HT, PGE2, TNF-α, IL-6, NF-κB, | - HP extract: ↓ LPS-induced 5-HT, PGE₂ (mouse colon) concentration-independent, comparable to harpagoside (12 μg/mL) and sulfasalazine (2 μg/mL)  - HP extract: ↓ TNF-α, IL-6, NF-κB gene expression dose-independent  - HP extract: ↓ LPS-induced PGE₂ release in HCT116 cells concentration-dependent | Recinella et al., 2020 (25) |

Abbreviations: 5-HT = 5-hydroxytryptamine (serotonin); 5-LOX = 5-lipoxygenase; ADAMTS-5 = A disintegrin and metalloproteinase with thrombospondin motifs-5; AKT = Protein kinase B; AP-1 = Activator protein 1; cAMP = Cyclic adenosine monophosphate; CB1/CB2 = Cannabinoid receptor 1/2; CD86 = Cluster of differentiation 86;

COX-1/COX-2 = Cyclooxygenase-1/2; CRP = C-reactive protein; DC = Ethanol extract of Harpagophytum procumbens; DCW = Devil’s Claw water extract; DCm = Metabolic extract of Harpagophytum procumbens; ELISA = Enzyme-linked immunosorbent assay; ERK = Extracellular signal-regulated kinase; FAAH = Fatty acid amide hydrolase; ICAM-1= Intercellular adhesion molecule 1; IFN-γ = Interferon gamma; IL-1β/IL-6/IL-8/IL-10/IL-12/IL-17A = Interleukin-1 beta/6/8/10/12/17A; iNOS = Inducible nitric oxide synthase; JAK2 = Janus kinase 2; L-SEL = L-selectin; LPS = Lipopolysaccharide; MAPK = Mitogen-activated protein kinase; MCP-1 = Monocyte chemoattractant protein 1; MMP-3/MMP-9/MMP-13 = Matrix metalloproteinase-3/9/13; NF-κB = Nuclear factor kappa-light-chain-enhancer of activated B cells; NO = Nitric oxide; PAI-1= Plasminogen activator inhibitor-1; PECAM-1 = Platelet endothelial cell adhesion molecule 1; PF-HR/CB = Phenylethanoid fractions of Harpagophytum root/cell biomass; PI3K = Phosphoinositide 3-kinase; PI-PLCβ2/γ1 = Phosphatidylinositol-specific phospholipase C beta 2 / gamma 1; PKA = Protein kinase A; p-ERK/p-PKA/p-cPLA2 = Phosphorylated ERK / PKA / cytosolic phospholipase A2; PGE₂ = Prostaglandin E₂; PSGL-1 = P-selectin glycoprotein ligand-1;

STAT1 = Signal transducer and activator of transcription 1; THP-1 = Human monocytic leukemia cell line; TNF-α = Tumor necrosis factor alpha; TPA = 12-O-tetradecanoylphorbol-13-acetate; TXB2 =Thromboxane B2; VCAM-1: = Vascular cell adhesion molecule 1

**Table S2. Anti-inflammatory activity of HP vs. NSAIDs: In vivo evidence**

| NSAID Comparison | Key comparator | Model/System | HP dose | Mechanism / Pathway | Key Findings Summary | Reference |
| --- | --- | --- | --- | --- | --- | --- |
| Yes | Indomethasin (2.5, 5, 10 mg/kg, i.p.) | Carrageenan induced paw edema | Water extract: 100, 200, 400 mg/kg Harpagoside: 5, 10 mg/kg, i.p. | - | - HP extract: ↓ carrageenan-induced paw edema dose-dependently (max 72% at 400 mg/kg, effect up to 24h)  - Harpagoside alone: no significant effect  - Indomethacin ↓ edema dose-dependently (max 58% at 10 mg/kg), comparable to HP extract | Lanhers et al., 1992 (26) |
| Yes | Indomethasin (10mg/kg, i.p.) | Carrageenan induced paw edema | Water extract: 400, 800, 1200 mg/kg i.p. | - | - HP extract: ↓ carrageenan-induced edema dose-dependently from 400 mg/kg (43% inhibition at 3h); max inhibition 53% (800 mg/kg) and 64% (1200 mg/kg) at 3h; effects sustained up to 5h  - Indomethacin 58% inhibition at 3h; effect maintained up to 24h, comparable to HP extract | Baghdikian et al., 1997 (27) |
| Yes | Diclofenac (100 mg/kg, i.p.) | Egg albumin induced paw edema | Water extract: 50–800 mg/kg i.p. | - | - HP extract: ↓ edema dose-dependently; 800 mg/kg < diclofenac efficacy | Mahomed & Ojewole, 2004 (28) |
| NO | - | Carrageenan induced paw edema | Wter extract, i.p.  Water extract +Cyclodextrin, p.o., intraduodenally injection: 200-1600 mg/kg | - | - HP extract (400 mg/kg): strong anti-inflammatory effect via i.p. (67%) and i.d. (60%) routes  - No anti-inflammatory activity via oral route at any dose  - Oral inefficacy: due to gastric acid degradation, not lack of pharmacological effect | Soulimani et al., 1994 (29) |
| No | - | Freund’s adjuvant induced arthritis | Ethanol extract: 25, 50, 100 mg/kg | - | - Acute: all doses ↓ paw edema, height, joint size; 50 & 100 mg/kg > 25 mg/kg; no effect on paw width  - Chronic: from day 29, progressive ↓ paw width, height, joint size (Days 24–40) | Andersen et al., 2004 (30) |
| No | - | TPA induced skin inflammation | Methanolic extract (DEV): 60, 300, 600 µg | COX-2 | - COX-2 inhibition: weak at 300 µg, present at 600 µg; no statistical analysis | Na et al., 2004 (5) |
| No | - | TPA induced skin inflammation | Methanol extract: 200, 400 µg | COX-1, COX-2, pERK, NF-κB, AP-1, c-Fos | - 400 µg: COX-2 ↓ 46%, COX-1 ↔  - ERK phosphorylation ↔, EIK phosphorylation ↓ (400 µg)  - NF-κB DNA binding & p65 nuclear translocation ↔  AP-1 DNA binding ↓ (400 µg) + c-Fos ↓ dose-dependently | Kundu et al., 2005 (31) |
| No | - | Carrageenan induced adrenalectomized | Organin solvent: 100–800 mg/kg i.p 800 mg/kg p.o. | - | - HP extract (i.p.): ↓ Inflammation & ↓ Leukocyte count  - Oral HP extract: No effect  - Anti-inflammatory effect: dose-dependent & adrenal-independent | Catelan et al., 2006 (32) |
| No | - | Surgically induced osteoarthritis | HP extract FB9195 (14% harpagoside), 150mg/day | TIMP-2 | - Demonstrated chondroprotective effects, significant improvement in elastin fiber structure  2–5 fold upregulation of TIMP-2 mRNA expression | Chrubasik et al., 2006 (33) |
| No | Prednisolone (10 mg/kg, p.o.) | Freund’s adjuvant induced arthritis | 50% ethanol: 50 mg/kg p.o. | - | - Oral HP extract: no effect in acute phase (day 3)  - Oral HP extract: anti-inflammatory effect in chronic phase (days 14 & 19)  - i.p. HP extract: Immediate anti-inflammatory effect (acute & chronic phases)  - Effect: dose-dependent & adrenal-independent | Inaba et al., 2010 (8) |
| No | - | Surgically induced osteoarthritis | HP extract (14% harpagoside), 150 mg/day, p.o. | - | - Slight protective trend in cartilage preservation  → 30% thickness & 26% volume reduction vs. control (35% & 38%)  → Differences not statistically significant | Wachsmuth et al., 2011 (34) |
| No | Apocynin (10 mg/kg, p.o.) | Arsenic induced neurotoxicity | DCW (Devil’s Claw Water Extract) capsules: 200, 400 mg/kg/day 21day, p.o. | CRP | - DCW (↑dose) → ↓serum CRP in arsenic-exposed rats (comparable to apocynin) | Peruru et al., 2020 (35) |

Abbreviations: HP = Harpagophytum procumbens; i.p. = Intraperitoneal injection; p.o. = oral administration; i.d. = Intraduodenal injection; TPA = 12-O-Tetradecanoylphorbol-13-acetate; COX-1/2 = Cyclooxygenase-1/2; ERK = Extracellular signal-regulated kinase; pERK = Phosphorylated ERK; NF-κB = Nuclear factor kappa-light-chain-enhancer of activated B cells; AP-1 = Activator protein 1; c-Fos = Cellular proto-oncogene Fos; TIMP-2 = Tissue inhibitor of metalloproteinase-2; DCW = Devil’s Claw water extract; CRP = C-reactive protein.

**Table S3. Anti-analgesic effect of HP vs. NSAIDs: In vivo evidence**

| NSAID comparison | Key comparator | Animal model | HP dose | Mechanism / Pathway | Key findings summary | Reference | |
| --- | --- | --- | --- | --- | --- | --- | --- |
| Yes | Acetylsalicylic acid (68 mg/kg, i.p.), morphine sulfate (1.15 mg/kg, i.p.) | Electrical heat, acetic acid-induced pain stimulus | Water extract: 100-400 mg/kg, Harpagoside 5-10 mg/mL, i.P. | - | - HP extract (↑dose): ↓writhings  - 100 mg/kg: 47% protection  - 400 mg/kg: 78% protection  - Likely due to harpagoside content | | Lanhers et al., 1992 (26) |
| Yes | Acetylsalicylic acid (68 mg/kg i.p.) | Acetic acid-induced pain stimulus | Water extract: 100, 400, 800 and 1200 mg/kg, i.P. | - | Significant analgesic effects from 400 mg/kg, comparable to or slightly greater than aspirin at 1200 mg/kg | | Baghdikian et al., 1997 (27) |
| Yes | Diclofenac (100 mg/kg i.p.) | Electrical heat, acetic acid-induced pain stimulus | Water extract: 50 - 800 mg/kg, i.p. | - | Dose-dependent analgesic effects (50–800 mg/kg, i.p.) in thermal and chemical pain models Toxicity (LD50): 1250 ± 156 mg/kg (i.p.) in mice – relatively safe dose range | | Mahomed et al., 2004 (28) |
| Yes | Naproxen (30 mg/kg i.p.) | Plantar incision or spared nerve injury | 70% ethanol extract: 300 mg/kg, p.o. | - | - Plantar incision + HP extract: ↓mechanical hyperalgesia (24 h)  - Plantar incision + HP extract: ↓22–27 kHz USVs (post-op pain indicator) | | Lim et al., 2014 (36) |
| No | Morphine (0.3 mg/kg, s.c.). | Formalin injected | Comercial extract: 100, 300 mg/kg p.o | Nitric Oxide (NO), opioid receptor-related mechanism | - Formalin test: ↓pain response (42.5%, 59.0%)  - Effect opioid receptor-mediated (blocked by naloxone)  - Tail-flick test → no significant effect | | Uchida et al., 2008 (37) |
| No | Morphine (3 mg/kg i.p.) | Chronic constriction injury | HP extract: 400, 600 and 800 mg/kg, i.p., 400 mg/kg + Morphine: 3 mg/kg, i.p. | HO-1/CO pathway activation | - HP extract (i.p., ↑dose) : ↑allodynic threshold (peak at 30–45 min)  - HP extract (400 mg/kg): ↓thermal hyperalgesia | | Parenti et al., 2016 (38) |
| No | - | Chronic arthritis model with freund's adjuvant | 60% ethanol extract: 25, 50, 100 mg/kg |  | - Acute & chronic administration: significant, sustained antinociception  - 100 mg/kg dose: maximal reduction in joint swelling & size (arthritis model) | | Andersen et al., 2004 (30) |
| No | - | Carrageenan-induced inflammatory pain | 70% ethanol extract: 300 and 800 mg/kg i.p. | HO-1/CO pathway activation | - HP extract (800 mg/kg, i.p.): ↓ mechanical allodynia (2–4 h) & thermal hyperalgesia (2–6 h)  - HP extract (300 mg/kg): no effect  - Effect: blocked by ZnPP IX (HO-1 inhibitor)  - Effect → restored by Hemin/CORM-3 → HO-1/CO pathway involvement. | | Parenti et al., 2015 (39) |
| No | - | Spinal cord injury | water extract: 300 mg/kg/day, p.o. |  | - SCI rats: ↓ mechanical thresholds  - HP extract: mitigated threshold reduction (from Day 15). | | Ungerer et al., 2020 (15) |
| No | - | Spinal lumber stenosis | water extract: 100, 200, 400 mg/kg, p.o. | TRPV1 pathway inhibition | - Dose-dependent ↓ TRPV1 expression  - ↑ withdrawal latency (von Frey mechanical test) | | Hong et al., 2022 (40) |

Abbreviations: CO = Carbon monoxide; CORM-3 = Carbon monoxide-releasing molecule-3; HO-1 = Heme oxygenase-1; HP = *Harpagophytum procumbens*; i.p. = Intraperitoneal injection; LD₅₀ =Median lethal dose; NO = Nitric oxide; p.o. = Oral administration; s.c. = Subcutaneous injection; SCI = Spinal cord injury; TRPV1 = Transient receptor potential vanilloid type 1; USVs = Ultrasonic vocalizations; ZnPP IX = Zinc protoporphyrin IX (HO-1 inhibitor).

**Table S4. Anti-oxidant effect of HP vs. NSAIDs: In vitro & ex vivo evidence**

| Related system | Cell/Tissue Type | Injury Inducer | HP Treatment | Assay Type | Key Findings Summary | Reference |
| --- | --- | --- | --- | --- | --- | --- |
| Immune/Inflammation | RAW 264.7,  HepG2 | LPS, 100 ng/mL | Harpagoside: 0.1 - 200 µM | NO, COX-2, iNOS | - HP extract: dose-dependent ↓ NO release in LPS-stimulated cells  - Harpagoside (200 µM): significant ↓ COX-2 & iNOS mRNA and protein expression (HepG2 cells). | Huang et al., 2006 (7) |
|  | RAW 264.7, Human neutrophil | LPS | HP commercial extract & tincture: 1, 10, 100, 1000 µg/mL (DPPH), 300, 500, 1000 µg/ml (NO, MPO) | DPPH, NO, MPO, SAG, | - 1000 µg/mL: 91.75% DPPH scavenging (IC₅₀ = 49.87 μg/mL)  - HP extract: dose-dependent NO inhibition  - Significant ↓ neutrophil SAG and suppression of neutrophil MPO activity | Grant et al., 2009 (41) |
|  | Human plasma | LPS, 100 μg/mL | 60% ethanol: 0.625-10 µg/mL Fractions A-C: 30, 100, 300 µg/mL | Total NO_2_^-^/NO_3_^-^ | - Fraction A (30 µg/mL): ↓ NO metabolites by 66% (p = 0.037) in LPS-stimulated whole blood  - Fraction C (30 µg/mL): ↓ NO metabolites by 67% (p = 0.005)  - NSAIDs (indomethacin, etoricoxib) → No effect on NO production  - Fraction B → No effect on NO production (similar to NSAIDs) | Anauate et al., 2010 (1) |
|  | Human neutrophils | PMA (25 nM), F. nucleatum (MOI 1:100), S. aureus (MOI 1:300) | 6 accessions of HP species, 5% (12.5 μg/mL) | ROS (luminol chemiluminescence) | - HP (5%): reduced HOCl and H₂O₂ generation (upon PMA or bacterial stimulation) | Muzila et al., 2016 (42) |
|  | Raw 264.7 | LPS, 1 µg/mL | commercial extract: 50, 100 μg/mL | NO | - Hp: significant ↓ NO• levels  - At 100 µg/mL after 24 h  - At 50 and 100 µg/mL after 6 days | Calabrese et al., 2021 (43) |
|  | BV-2 | LPS, 100 ng/mL | water extract: 10, 20, 40 80 μg/mL | NO, ROS (DCFH-DA), NRF2, HO-1 | - HP extract: dose-dependent ↓ LPS-induced NO and ROS production  - HP extract: dose-dependent ↑ Nrf2 and HO-1 expression (not statistically significant) | Ungerer et al., 2020 (15) |
|  | THP-1, HMEC-1 | TNFα, 10 ng/mL | Flonat Fast®: 250 μg/mL | DCF-DA | - Flonat Fast®: ↓ TNF-α-induced intracellular ROS production  - 25% reduction in THP-1 cells (p < 0.05)  - 21% reduction in HMEC-1 cells | Quarta et al., 2022 (19) |
|  | BV-2 | LPS, 0.1 µg/mL | 70% ethanol extrat ethyl acetate fraction: 50, 100, 200 µg/mL | ROS (DCFH-DA) assay | - Significant ↓ LPS-induced ROS at 100 µg/mL; no effect at 50 or 200 µg/mL | Lima et al., 2023 (44) |
| Gastrointestinal/Colon | Human colorectal mucosal biopsies | - | water extract: 1:50-1:500,000 | Xanthine oxidase, Phycoerythrin degradation cell-free assay | Dose-dependent xanthine oxidase and peroxyl radical scavenging activity | Langmead et al., 2002 (45) |
|  | HCT116, Rat colon tissue | H_2_O_2_, 1mM | Microwave water extracts: 100, 500, 1000 µg/mL | ROS (DCFH-DA), 8-iso-PGF2α | Significantly reduced H₂O₂-induced ROS generation in HCT116 cells and LPS-induced 8-iso-PGF₂α levels in a dose-dependent manner in rat colon tissue. | Locatelli et al., 2017 (24) |
|  | Mouse colon specimen | LPS, 10 μg/mL | Water extract: 100, 500, 1000 µg/mL Harpagoside: 12 µg/mL | 8-iso-PGF2α, Nrf2, DPPH, β-carotene/linoleic acid | Reduced LPS-induced 8-iso-PGF₂α, Nrf2, DPPH activity; showed antioxidant activity comparable to Trolox. | Recinella et al., 2020 (25) |
| Neural tissues | Rat brain homogenate | SNP, 5 µM and Fe²⁺, 100 µM | Crude extract, infusion, Ethyl acetate, n-butanol, Chloroform fractions, 0.5 - 2000 µg/mL | TBARS, Catalase, Thiol levels | Ethyl acetate fraction dose-dependently reduced Fe²⁺-induced TBARS and prevented catalase depletion and thiol oxidation, comparable to gallic acid | Schaffer et al., 2013 (46) |
|  | Frontal&parietal cortex synaptosome | Amyloid β-peptide (1–40) , 1 μM | Microwave water extracts: 10, 25, 50 μg/mL | MDA, 3-HK | Significantly reduced amyloid β-induced MDA and 3-HK levels in cortical synaptosomes of both young and aged rats | Ferrante et al., 2017 (47) |
|  | Primary spinal cord neurons | FeSO_4_, 50 µM | water extract: 50, 100, 200 mg/kg | Ferritin, TfR, NRF2 | Dose-dependent reduction in Ferritin, TfR, and NRF2 expression | Hong et al., 2022 (40) |
| Parenchymal cells | L929 | LPS, 5 μg/mL | Water ectract: 0.1, 1 mg/mL | iNOS, NO | Suppressed LPS-induced iNOS mRNA expression and NO production in a dose-dependent manner | Jang et al., 2003 (3) |
|  | Rat mesangial cell | IL-1β, 0.5 nM | Extract1-5 | NO, iNOS | - Extracts 1 (8.9% harpagoside) and 2 (27% harpagoside): dose-dependent inhibition of IL-1β-induced NO production and iNOS expression  - Extract 2 (27% harpagoside): greater potency than Extract 1. | Kaszkin et al., 2004 (4) |
| Plant-derived cells | In vitro-derived Devil’s claw cell suspension (CME-CS) and transformed root cultures (CME-HR) | - | Crude methanolic extracts and Phenylethanoid-rich fractions: 250, 500, 1000 µg/mL | FRAP, Fe^2+^-chelating activity | - FRAP assay: verbascoside exhibited highest antioxidant activity  - Fe²⁺-chelating activity: detected only in methanolic crude extracts, 1.5–2× higher than butylated hydroxyanisole | Georgiev et al., 2012 (48) |
| Cell-free assays | Cell-free DPPH assay | - | Methanol extract | DPPH, Oxygen radical absorbance capacity (ORAC), superocide anion | PF extract: demonstrated potent antioxidant activity  β-OH-verbascoside: highest DPPH scavenging (IC₅₀ = 0.12 mg/mL)  Leucosceptoside A: strongest ORAC activity (>20,000 units/g)  PF: significant superoxide scavenging (IC₅₀ = 5.5 μg/mL)  SF & TF: no significant antioxidant activity | Grąbkowska et al., 2016 (49) |
|  | Cell-free DPPH assay | - | 70% ethanol extract ethyl acetate fraction: 50, 100, 150, 200, 250 μg/mL | DPPH | - Ethyl acetate fraction: Strongest DPPH scavenging activity  - IC₅₀ = 38.21 ± 7.95 μg/mL | Schaffer et al., 2016 (50) |

Abbreviations: 3-HK = 3-Hydroxykynurenine; 8-iso-PGF₂α = 8-iso-Prostaglandin F₂ alpha; AP-1 = Activator protein 1; BV-2 = Mouse microglial cell line; Catalase = Hydrogen peroxide-decomposing enzyme; CME-CS: Cell suspension culture extract of *Harpagophytum procumbens;* CME-HR = Hairy root culture extract of *Harpagophytum procumbens;* DCF-DA = 2′,7′-Dichlorodihydrofluorescein diacetate (ROS detection dye); DPPH = 2,2-Diphenyl-1-picrylhydrazyl (free radical scavenging assay); Fe²⁺ = Ferrous ion; FRAP = Ferric reducing antioxidant power; H₂O₂ = Hydrogen peroxide; HCT116 = Human colorectal carcinoma cell line; HO-1 = Heme oxygenase-1; HP = *Harpagophytum procumbens;* iNOS = Inducible nitric oxide synthase; IL-1β = Interleukin-1 beta; L929 = Mouse fibroblast-like cell line; LPS = Lipopolysaccharide; MDA = Malondialdehyde (lipid peroxidation marker); MPO = Myeloperoxidase; NO = Nitric oxide; NO₂⁻/NO₃⁻ = Nitrite/nitrate (stable NO metabolites); NRF2 = Nuclear factor erythroid 2–related factor 2; ORAC = Oxygen radical absorbance capacity; PMA = Phorbol 12-myristate 13-acetate; ROS = Reactive oxygen species; SAG = Soluble antigen; SNP = Sodium nitroprusside (NO donor); TBARS = Thiobarbituric acid reactive substances (lipid peroxidation assay); TfR = Transferrin receptor; THP-1 = Human monocytic leukemia cell line; TNF-α = Tumor necrosis factor alpha; Trolox = Water-soluble vitamin E analog (standard antioxidant control).

**Table S5. Anti-oxidant effect of HP vs. NSAIDs: In vivo evidence**

| NSAID Comparison | Key comparator | Animal model | HP dose | Mechanism / Pathway | Key Findings Summary | Reference |
| --- | --- | --- | --- | --- | --- | --- |
| Yes | Ibuprofen (20 mg/kg) | Spinal cord contusion injury | water extract: 300 mg/kg, p.o. | 4-HNE, 4-HHE, HO-1, NQO1 | - Post-SCI HP treatment: ↓ 4-HNE, ↑ 4-HHE, normalized 4-HNE/4-HHE ratio  -↑HO-1 expression  - Non-significant ↑ in Nrf2 expression | Ungerer et al., 2020 (15) |
| No | - | Formalin injected | 300 mg/kg p.o. | Nitrites/Nitrates (NOx) | Reduced formalin-induced NOₓ levels in the spinal cord | Uchida et al., 2008 (37) |
| No | - | Formalin-induced knee joint cartilage damage | 23 mg/kg p.o. | MDA, NO, 8-OH/Gua, tGSH | - GCMHB mixture (include HP, 20 mg/kg): ↓ MDA and NO → attenuated formalin-induced oxidative stress in rats. | Ucuncu et al., 2015 (51) |
| No | - | Fluphenazine-induced orofacial dyskinesia | 70% ethanol extract ethyl acetate fraction: 10, 30, 100 mg/kg i.p. | TBARS, DCFH-DA, Catalase activity, Protein and non-protein thiol levels) | - EAF HP (ethyl acetate fraction) alone: no effect on oxidative stress markers in any tissue  - In brain: ↓ fluphenazine-induced oxidative stress. | Schaffer et al., 2016 (50) |
| No | Apocynin (10 mg/kg) | Arsenic-induced neurotoxicity | 200, 400 mg/kg p.o. | GSH, SOD, Catalase, MDA, NO | - Dose-dependent ↑ GSH, SOD, CAT; ↓ MDA, NO  - In arsenic-exposed animals: significant antioxidant effect (comparable to apocynin) | Peruru et al., 2020 (35) |
| No | - | Silicone-induced spinal stenosis | water extract: 100, 200, 400 mg/kg p.o. | iNOS, COX-2, Ferritin, NRF2 | - Dose-dependent ↓ iNOS & ferritin; ↑ NRF2 activity in spinal cord  - COX-2 ↓ observed only at 400 mg/kg | Hong et al., 2022 (40) |
| No | - | Acetic acid-induced colitis | 50 mg/kg p.o. | GSH, SOD, Catalase, MDA | - Lycopodium: ↑ GSH, SOD, CAT; ↓ MDA in IBD-induced colon tissue  - Effects were time- and treatment-dependent | Bastaki et al., 2022 (52) |
| No | - | Amphetamine-induced psychosis | 70% ethanol extrat ethyl acetate fraction: 30 mg/kg i.p | DCFH-DA assay | - ↑ Protein thiol levels in cortex (p < 0.05)  - No significant effect on DCFH-measured ROS or in striatum | Lima et al., 2023 (44) |

Abbreviations: 4-HHE = 4-Hydroxyhexenal; 4-HNE = 4-Hydroxynonenal; CAT = Catalase; COX-2 = Cyclooxygenase-2; DCFH-DA = 2′,7′-Dichlorodihydrofluorescein diacetate (ROS indicator); EAF = Ethyl acetate fraction; GCMHB = Combination of Ginkgo biloba, Crocus sativus, Melissa officinalis, Harpagophytum procumbens, and Boswellia serrata; GSH = Glutathione; HP = Harpagophytum procumbens; HO-1 = Heme oxygenase-1; i.n. = Intranasal administration; iNOS = Inducible nitric oxide synthase; i.p. = Intraperitoneal injection; MDA = Malondialdehyde; NO = Nitric oxide; Nox = Nitrites/Nitrates (combined indicator of NO levels) NRF2 = Nuclear factor erythroid 2–related factor 2; NQO1 = NAD(P)H quinone dehydrogenase 1; p.o. = Oral administration; ROS = Reactive oxygen species; SCI = Spinal cord injury; SOD = Superoxide dismutase; TBARS = Thiobarbituric acid reactive substances; tGSH = Total glutathione; 8-OH/Gua = 8-Hydroxyguanosine (marker of oxidative DNA damage).

**Table S6. Cryoprotective effect of HP vs. NSAIDs: In vitro & ex vivo evidence**

| Related System | Cell Type | Injury Inducer | HP Treatment | Assay Type | Main Findings | Reference |
| --- | --- | --- | --- | --- | --- | --- |
| Inflammation | L929 | - | 0.001 - 1 mg/mL | MTT | - No significant cytotoxicity  - Cell viability ≥89%; peaked at 105% at 1 mg/mL | Jang et al., 2003 (3) |
|  | RAW 264.7, Human neutrophils | LPS, fMLP, AA | 300, 500, 1000 µg/ml | Trypan blue | - LPS-stimulated RAW 264.7 cells : >93% viability after HP treatment; no cytotoxicity  - fMLP- and AA-induced neutrophils: >93% viability at all doses; no significant difference vs. control. | Grant et al., 2009 (41) |
|  | RAW 264.7 | LPS, 1 µg/ml | HP: 50, 200, 500 µg/mL Harpagoside: 50, 100, 200 µM | TetraColor | - No cytotoxicity up to 500 µg/mL (HP) or 200 µM (Harpagoside) | Inaba et al., 2010 (8) |
|  | Human neutrophils | - | 5, 10, 50, 100% (= 12.5, 25, 125, 250 µg/ml) | ATP | - No cytotoxicity among 5–100% HP | Muzila et al., 2016 (42) |
|  | RAW 264.7, Human chondrocyte | - | RAW 264.7: 50, 100, 200 μg/mL Human chondrocyte: 50, 100 μg/mL | MTT | - RAW 264.7 cells:  24 h: No cytotoxicity at 50–200 µg/mL; slight proliferation observed  6 days: Significant dose-dependent cytotoxicity  - Human chondrocytes: No cytotoxicity at 24 h and 6 days | Calabrese et al., 2021 (43) |
|  | THP-1, HMEC-1 | TNF-α, 10 ng/ml | 250, 500 μg/mL | MTT | - THP-1 and HMEC-1: No cytotoxicity at 250 and 500 μg/mL HP | Quarta et al., 2022 (19) |
|  | BV-2 | LPS, 0.1 µg/ml | 10 - 500 µg/ml | MTT | - 300 μg/mL reduced viability <50% at 72 h (cytotoxic) | Lima et al., 2023 (44) |
| Nerve/CNS tissue | Rat brain cortical slices | SNP, 10 µM or Fe^2+^, 200 µM | 100, 200, 400 µg/ml | MTT | - Fe²⁺-induced damage: Significant increase in viability at 100–400 µg/mL - SNP-induced damage: Significant increase in viability at 400 µg/mL | Schaffer et al., 2013 (46) |
|  | Hypo-E22 | - | 10–200 μg/mL | MTT | - No cytotoxicity at 10–50 μg/mL; significant decrease in viability at 100–200 μg/mL. | Ferrante et al., 2017 (47) |
|  | Primary spinal cord neurons | FeSO_4_, 50 µM | CCK: 10, 25, 50, 100, 200 μg/mL Other assays: 50, 100, or 200 μg/mL | 1) CCK 2) Live/Dead assay 3) Annexin V/PI apoptosis | - No cytotoxicity up to 200 μg/mL; Significant, dose-dependent ↑ cell viability against FeSO₄ toxicity  - ↓ PI⁺ necrotic cells to 14.6% at 100–200 µg/mL vs. 23.7% (FeSO₄ control) | Hong et al., 2022 (40) |
| Bone | Mouse BMMs | M-CSF, 30 ng/mL | 25, 50, 100 µM | XTT | - No cytotoxicity during osteoclast differentiation with all doses. | Kim et al., 2015 (53) |
|  | MC3T3-E1, BMCs | - | Harpagide: 0.032 - 500 μM (MC3T3-E1), 0.032 - 100 μM (BMCs) | MTT | - MC3T3-E1 cells: No cytotoxicity up to 500 μM harpagide; >90% viability - Osteoclast differentiation from BMCs: No cytotoxicity up to 4 μM harpagide; >85% cell viability | Chung et al., 2016 (54) |
|  | MC3T3-E1, BMCs | - | Harpagoside: 0.032 - 500 μM (MC3T3-E1), 0.032 - 100 μM (BMCs) | MTT | - MC3T3-E1 cells: No cytotoxicity up to 4 μM; >90% viability - Osteoclast differentiation from BMCs: No cytotoxicity up to 4 μM; >85% cell viability | Chung et al., 2017 (55) |
| Cancer | HCT116 | - | 100, 500, 1000 µg/ml | MTT | - Significant reduction of viability at 1000 μg/mL | Locatelli et al., 2017 (24) |
|  | HCT116 | LPS, 10 µg/ml | 100 - 1000 µg/ml | MTT | - LPS reduced viability at 48 h, not at 24 h - HP (1000 μg/mL) decreased viability at both time points. | Recinella et al., 2020 (25) |
| Microbial / DNA repair | E.coli AB1157, BW110, BH110 | - | 40 g/mL | Bacterial survival fraction | - No cytotoxicity with HP aqueous extract; survival unaffected regardless of DNA repair capacity. | Almeida et al., 2007 (56) |
| Kidney/obesity | Hek293a | - | HP: 0.125 - 10 mg/mL Harpagoside: 0.125 - 1 mg/mL | Resazurin | - No cytotoxicity after 4 h exposure up to 10 mg/mL (HP) or 1 mg/mL (Harpagoside) | Torres-Fuentes et al., 2014 (57) |
| Muscle | C2C12 | - | 100, 500, 1000 µg/ml | MTT | - No cytotoxicity up to 1000 μg/mL | Locatelli et al., 2017 (24) |

Abbreviations: AA = Arachidonic acid; Annexin V/PI = Annexin V and Propidium Iodide (used in apoptosis detection); ATP assay = Adenosine triphosphate–based luminescence cytotoxicity assay; BMCs = Bone marrow cells; BMMs = Bone marrow–derived macrophages; CCK = Cell Counting Kit; CNS = Central nervous system; DCF-DA = 2′,7′-Dichlorofluorescin diacetate (ROS-sensitive fluorescent dye); E. coli = Escherichia coli; Fe²⁺ = Ferrous ion; fMLP = N-Formylmethionyl-leucyl-phenylalanine (neutrophil activator); HCT116 = Human colorectal carcinoma cell line; HEK293a = Human embryonic kidney 293 cells; HMEC-1 = Human microvascular endothelial cell line; HP = Harpagophytum procumbens; LPS = Lipopolysaccharide; MC3T3-E1 = Mouse calvaria-derived pre-osteoblastic cell line; MTT = 3-(4,5-dimethylthiazol-2-yl)-2,5-diphenyltetrazolium bromide (cell viability assay); NO = Nitric oxide; RAW 264.7 = Mouse macrophage-like cell line; ROS = Reactive oxygen species; SNP = Sodium nitroprusside (NO donor compound); THP-1 = Human monocytic leukemia cell line; TNF-α = Tumor necrosis factor alpha; Trypan blue = Vital dye for cell viability (exclusion assay); XTT = Sodium 3′-[1-(phenylaminocarbonyl)-3,4-tetrazolium]-bis(4-methoxy-6-nitro) benzene sulfonic acid hydrate (cell viability assay);

**Table S7. Summary of clinical efficacy outcomes for HP**

| Intervention | Study design | Sample size (n) | Follow-up duration (weeks) | VAS Pain Score (0-10 or 0-100mm) | WOMAC Total Score | Secondary Endpoints | Rescue Medication Usage | Reference |
| --- | --- | --- | --- | --- | --- | --- | --- | --- |
| HP extract LI 174: 960mg/day (480 mg twice daily) | Single-arm | 117 | 8 | - | - | • Arhus total Index improvement:  8wk: 5.9 → 2.1  (-64.4%)  • Multidimensional Pain Scale:  8wk: 10.7 → 4.7  (-56.1%)  • Finger-Floor Distnace:  8wk: 15.1 cm → 10.2 cm (32.5%)  • Clinical Global Impression:  8wk: 4.6 → 2.9  (-37.0%) | - | Laudahn & Walper., 2001 (58) |
| Doloteffin®: 2400mg/day (harpagoside 50mg) | Single-arm | 75 | 12 | • Current pain: 5.90 → 4.38 (-25.8%)  • Total pain index: 6.48 → 4.89 (-24.5%) | • Total score: 4.77 → 3.51 (-26.4%) | - | - | Wegener et al., 2003 (59) |
| HP extract: 960mg/day (480 mg twice daily) | Single-arm | 259 (ITT: 222) | 8 | - | • Total score: 4.6±1.5 → 2.9±1.8 (-37.0%) | • NRS Pain (8wk):   4.9±2.1 → 3.3±2.2 (-32.7%)  • NRS Stiffness (8wk):  8wk: 5.1±2.3 → 3.1±2.2  (-39.2%)  • NRS Function (8wk):  3.5±2.5 → 2.1±2.3  (-40.0%) | - | Warnock et al., 2007 (60) |
| Artipotect® (included with HP extract 150 mg) | Single-arm, OL | 130 | 12, 24 | • 12 wk: 6.28±1.68 → 4.03±2.02 (-35.8%)  • 24 wk: 6.28±1.68 → 2.58±1.72 (-58.9%) | • 12 wk: 43.24±21.51 → 30.60±18.04 (-29.2%)  • 24 wk: 43.24±21.51 → 20.76±15.73 (-52.0%) | - | • Lequesne 12 wk: 11.11±5.33 → 7.56±4.40 (-33.7%) 24 wk: 11.11±5.33 → 4.81±3.68 (-57.8%) | Puigdellivol et al., 2019 (61) |
| Tregocel® (included with HP extract 1000 mg) | Single-arm, OL | 137 | 36 | • 12 wk: 60.0 → 37.0 (-38.3%)  • 24 wk: 60.0 → 27.0 (-55.0%)  • 36 wk: 60.0 → 21.0 (-65.0%) | - | - | • 6-minute walk test:  382.8±88.1m → 408.8±96.3m • Analgesic reduction:  36 wk: 99.1% → 55.1% (-44.4% usage) | Żęgota et al., 2021 (62) |
| Artipotect Forte®: (included with HP extract 150 mg) | Single-arm, OL | 186 | 12, 24 | • 12 wk: 6.18±1.59 → 4.19±1.75 (-32.2%)  • 24 wk: 6.18±1.59 → 2.63±1.69 (-57.4%) | • 12 wk: 70.40±20.65 → 56.58±18.02 (-19.6%)  • 24 wk: 70.40±20.65 → 44.86±17.21 (-36.3%) | - | • Lequesne Index:  12 wk: 11.67±4.52 → 7.73±4.18 (-33.7%)  • 24 wk: 11.67±4.52 → 4.92±4.53 (-57.8%) | Puigdellívol et al., 2024 (63) |

Abbreviations: VAS = Visual analogue scale; WOMAC = Western ontario and mcMaster universities osteoarthritis index; OL = Open-label; HP = Harpagophytum procumbens; NRS = Numeric rating scale.

**Reference**

1. Anauate MC, Torres LM, de Mello SB. Effect of isolated fractions of Harpagophytum procumbens D.C. (devil's claw) on COX-1, COX-2 activity and nitric oxide production on whole-blood assay. Phytotherapy research : PTR. 2010;24(9):1365-9.

2. Fiebich BL, Heinrich M, Hiller KO, Kammerer N. Inhibition of TNF-alpha synthesis in LPS-stimulated primary human monocytes by Harpagophytum extract SteiHap 69. Phytomedicine. 2001;8(1):28-30.

3. Jang MH, Lim S, Han SM, Park HJ, Shin I, Kim JW, et al. Harpagophytum procumbens suppresses lipopolysaccharide-stimulated expressions of cyclooxygenase-2 and inducible nitric oxide synthase in fibroblast cell line L929. J Pharmacol Sci. 2003;93(3):367-71.

4. Kaszkin M, Beck KF, Koch E, Erdelmeier C, Kusch S, Pfeilschifter J, et al. Downregulation of iNOS expression in rat mesangial cells by special extracts of Harpagophytum procumbens derives from harpagoside-dependent and independent effects. Phytomedicine. 2004;11(7-8):585-95.

5. Na HK, Mossanda KS, Lee JY, Surh YJ. Inhibition of phorbol ester-induced COX-2 expression by some edible African plants. Biofactors. 2004;21(1-4):149-53.

6. Günther M, Laufer S, Schmidt PC. High anti-inflammatory activity of harpagoside-enriched extracts obtained from solvent-modified super- and subcritical carbon dioxide extractions of the roots of Harpagophytum procumbens. Phytochem Anal. 2006;17(1):1-7.

7. Huang TH, Tran VH, Duke RK, Tan S, Chrubasik S, Roufogalis BD, et al. Harpagoside suppresses lipopolysaccharide-induced iNOS and COX-2 expression through inhibition of NF-kappa B activation. Journal of ethnopharmacology. 2006;104(1-2):149-55.

8. Inaba K, Murata K, Naruto S, Matsuda H. Inhibitory effects of devil's claw (secondary root of Harpagophytum procumbens) extract and harpagoside on cytokine production in mouse macrophages. J Nat Med. 2010;64(2):219-22.

9. Gyurkovska V, Alipieva K, Maciuk A, Dimitrova P, Ivanovska N, Haas C, et al. Anti-inflammatory activity of Devil’s claw in vitro systems and their active constituents. Food Chemistry. 2011;125(1):171-8.

10. Fiebich BL, Muñoz E, Rose T, Weiss G, McGregor GP. Molecular targets of the antiinflammatory Harpagophytum procumbens (devil's claw): inhibition of TNFα and COX-2 gene expression by preventing activation of AP-1. Phytotherapy research : PTR. 2012;26(6):806-11.

11. Hostanska K, Melzer J, Rostock M, Suter A, Saller R. Alteration of anti-inflammatory activity of Harpagophytum procumbens (devil's claw) extract after external metabolic activation with S9 mix. J Pharm Pharmacol. 2014;66(11):1606-14.

12. Kim TK, Park KS. Inhibitory effects of harpagoside on TNF-α-induced pro-inflammatory adipokine expression through PPAR-γ activation in 3T3-L1 adipocytes. Cytokine. 2015;76(2):368-74.

13. Schopohl P, Grüneberg P, Melzig MF. The influence of harpagoside and harpagide on TNFα-secretion and cell adhesion molecule mRNA-expression in IFNγ/LPS-stimulated THP-1 cells. Fitoterapia. 2016;110:157-65.

14. Haseeb A, Ansari MY, Haqqi TM. Harpagoside suppresses IL-6 expression in primary human osteoarthritis chondrocytes. J Orthop Res. 2017;35(2):311-20.

15. Ungerer G, Cui J, Ndam T, Bekemeier M, Song H, Li R, et al. Harpagophytum procumbens Extract Ameliorates Allodynia and Modulates Oxidative and Antioxidant Stress Pathways in a Rat Model of Spinal Cord Injury. Neuromolecular Med. 2020;22(2):278-92.

16. Mariano A, Di Sotto A, Leopizzi M, Garzoli S, Di Maio V, Gullì M, et al. Antiarthritic Effects of a Root Extract from Harpagophytum procumbens DC: Novel Insights into the Molecular Mechanisms and Possible Bioactive Phytochemicals. Nutrients. 2020;12(9).

17. Koycheva IK, Mihaylova LV, Todorova MN, Balcheva-Sivenova ZP, Alipieva K, Ferrante C, et al. Leucosceptoside A from Devil's Claw Modulates Psoriasis-like Inflammation via Suppression of the PI3K/AKT Signaling Pathway in Keratinocytes. Molecules. 2021;26(22).

18. Mariano A, Bigioni I, Mattioli R, Di Sotto A, Leopizzi M, Garzoli S, et al. Harpagophytum procumbens Root Extract Mediates Anti-Inflammatory Effects in Osteoarthritis Synoviocytes through CB2 Activation. Pharmaceuticals (Basel). 2022;15(4).

19. Quarta S, Santarpino G, Carluccio MA, Calabriso N, Scoditti E, Siculella L, et al. Analysis of the Anti-Inflammatory and Anti-Osteoarthritic Potential of Flonat Fast(®), a Combination of Harpagophytum Procumbens DC. ex Meisn., Boswellia Serrata Roxb., Curcuma longa L., Bromelain and Escin (Aesculus hippocastanum), Evaluated in In Vitro Models of Inflammation Relevant to Osteoarthritis. Pharmaceuticals (Basel). 2022;15(10).

20. Mariano A, Ammendola S, Migliorini A, Leopizzi M, Raimondo D, Scotto d'Abusco A. Intron retention in PI-PLC γ1 mRNA as a key mechanism affecting MMP expression in human primary fibroblast-like synovial cells. Cell Biochem Funct. 2024;42(5):e4091.

21. Abdelouahab N, Heard C. Effect of the major glycosides of Harpagophytum procumbens (Devil's Claw) on epidermal cyclooxygenase-2 (COX-2) in vitro. J Nat Prod. 2008;71(5):746-9.

22. Ouitas NA, Heard CM. A novel ex vivo skin model for the assessment of the potential transcutaneous anti-inflammatory effect of topically applied Harpagophytum procumbens extract. International journal of pharmaceutics. 2009;376(1-2):63-8.

23. Ouitas NA, Heard C. Estimation of the relative antiinflammatory efficacies of six commercial preparations of Harpagophytum procumbens (Devil's Claw). Phytotherapy research : PTR. 2010;24(3):333-8.

24. Locatelli M, Ferrante C, Carradori S, Secci D, Leporini L, Chiavaroli A, et al. Optimization of Aqueous Extraction and Biological Activity of Harpagophytum procumbens Root on Ex Vivo Rat Colon Inflammatory Model. Phytotherapy research : PTR. 2017;31(6):937-44.

25. Recinella L, Chiavaroli A, Ronci M, Menghini L, Brunetti L, Leone S, et al. Multidirectional Pharma-Toxicological Study on Harpagophytum procumbens DC. ex Meisn.: An IBD-Focused Investigation. Antioxidants (Basel). 2020;9(2).

26. Lanhers MC, Fleurentin J, Mortier F, Vinche A, Younos C. Anti-inflammatory and analgesic effects of an aqueous extract of Harpagophytum procumbens. Planta Med. 1992;58(2):117-23.

27. Baghdikian B, Lanhers MC, Fleurentin J, Ollivier E, Maillard C, Balansard G, et al. An analytical study, anti-inflammatory and analgesic effects of Harpagophytum procumbens and Harpagophytum zeyheri. Planta Med. 1997;63(2):171-6.

28. Mahomed IM, Ojewole JA. Analgesic, antiinflammatory and antidiabetic properties of Harpagophytum procumbens DC (Pedaliaceae) secondary root aqueous extract. Phytotherapy research : PTR. 2004;18(12):982-9.

29. Soulimani R, Younos C, Mortier F, Derrieu C. The role of stomachal digestion on the pharmacological activity of plant extracts, using as an example extracts of Harpagophytum procumbens. Can J Physiol Pharmacol. 1994;72(12):1532-6.

30. Andersen ML, Santos EH, Seabra Mde L, da Silva AA, Tufik S. Evaluation of acute and chronic treatments with Harpagophytum procumbens on Freund's adjuvant-induced arthritis in rats. Journal of ethnopharmacology. 2004;91(2-3):325-30.

31. Kundu JK, Mossanda KS, Na HK, Surh YJ. Inhibitory effects of the extracts of Sutherlandia frutescens (L.) R. Br. and Harpagophytum procumbens DC. on phorbol ester-induced COX-2 expression in mouse skin: AP-1 and CREB as potential upstream targets. Cancer Lett. 2005;218(1):21-31.

32. Catelan SC, Belentani RM, Marques LC, Silva ER, Silva MA, Caparroz-Assef SM, et al. The role of adrenal corticosteroids in the anti-inflammatory effect of the whole extract of Harpagophytum procumbens in rats. Phytomedicine. 2006;13(6):446-51.

33. Chrubasik JE, Lindhorst E, Neumann E, Gerlach U, Faller-Marquardt M, Torda T, et al. Potential molecular basis of the chondroprotective effect of Harpagophytum procumbens. Phytomedicine. 2006;13(8):598-600.

34. Wachsmuth L, Lindhorst E, Wrubel S, Hadzhiyski H, Hudelmaier M, Eckstein F, et al. Micro-morphometrical assessment of the effect of Harpagophytum procumbens extract on articular cartilage in rabbits with experimental osteoarthritis using magnetic resonance imaging. Phytotherapy research : PTR. 2011;25(8):1133-40.

35. Peruru R, Usha Rani R, Thatiparthi J, Sampathi S, Dodoala S, Prasad K. Devil's claw (Harpagophytum procumbens) ameliorates the neurobehavioral changes and neurotoxicity in female rats exposed to arsenic. Heliyon. 2020;6(5):e03921.

36. Lim DW, Kim JG, Han D, Kim YT. Analgesic effect of Harpagophytum procumbens on postoperative and neuropathic pain in rats. Molecules. 2014;19(1):1060-8.

37. Uchida S, Hirai K, Hatanaka J, Hanato J, Umegaki K, Yamada S. Antinociceptive effects of St. John's wort, Harpagophytum procumbens extract and Grape seed proanthocyanidins extract in mice. Biol Pharm Bull. 2008;31(2):240-5.

38. Parenti C, Aricò G, Pennisi M, Venditti A, Scoto GM. Harpagophytum procumbens extract potentiates morphine antinociception in neuropathic rats. Nat Prod Res. 2016;30(11):1248-55.

39. Parenti C, Aricò G, Chiechio S, Di Benedetto G, Parenti R, Scoto GM. Involvement of the Heme-Oxygenase Pathway in the Antiallodynic and Antihyperalgesic Activity of Harpagophytum procumbens in Rats. Molecules. 2015;20(9):16758-69.

40. Hong JY, Kim H, Lee J, Jeon WJ, Lee YJ, Ha IH. Harpagophytum procumbens Inhibits Iron Overload-Induced Oxidative Stress through Activation of Nrf2 Signaling in a Rat Model of Lumbar Spinal Stenosis. Oxid Med Cell Longev. 2022;2022:3472443.

41. Grant L, McBean DE, Fyfe L, Warnock AM. The inhibition of free radical generation by preparations of Harpagophytum procumbens in vitro. Phytotherapy research : PTR. 2009;23(1):104-10.

42. Muzila M, Rumpunen K, Wright H, Roberts H, Grant M, Nybom H, et al. Alteration of Neutrophil Reactive Oxygen Species Production by Extracts of Devil's Claw (Harpagophytum). Oxid Med Cell Longev. 2016;2016:3841803.

43. Calabrese G, Zappalà A, Dolcimascolo A, Acquaviva R, Parenti R, Malfa GA. Phytochemical Analysis and Anti-Inflammatory and Anti-Osteoarthritic Bioactive Potential of Verbascum thapsus L. (Scrophulariaceae) Leaf Extract Evaluated in Two In Vitro Models of Inflammation and Osteoarthritis. Molecules. 2021;26(17).

44. Lima VB, Viana AR, Santos D, Felipetto N, Mezzomo NF, Zago AM, et al. Ethyl Acetate Fraction of Harpagophytum procumbens Prevents Oxidative Stress In Vitro and Amphetamine-Induced Alterations in Mice Behavior. Neurochem Res. 2023;48(6):1716-27.

45. Langmead L, Dawson C, Hawkins C, Banna N, Loo S, Rampton DS. Antioxidant effects of herbal therapies used by patients with inflammatory bowel disease: an in vitro study. Aliment Pharmacol Ther. 2002;16(2):197-205.

46. Schaffer LF, Peroza LR, Boligon AA, Athayde ML, Alves SH, Fachinetto R, et al. Harpagophytum procumbens prevents oxidative stress and loss of cell viability in vitro. Neurochem Res. 2013;38(11):2256-67.

47. Ferrante C, Recinella L, Locatelli M, Guglielmi P, Secci D, Leporini L, et al. Protective Effects Induced by Microwave-Assisted Aqueous Harpagophytum Extract on Rat Cortex Synaptosomes Challenged with Amyloid β-Peptide. Phytotherapy research : PTR. 2017;31(8):1257-64.

48. Georgiev MI, Alipieva K, Orhan IE. Cholinesterases inhibitory and antioxidant activities of Harpagophytum procumbens from in vitro systems. Phytotherapy research : PTR. 2012;26(2):313-6.

49. Grąbkowska R, Matkowski A, Grzegorczyk-Karolak I, Wysokińska H. Callus cultures of Harpagophytum procumbens (Burch.) DC. ex Meisn.; production of secondary metabolites and antioxidant activity. South African Journal of Botany. 2016;103:41-8.

50. Schaffer LF, de Freitas CM, Chiapinotto Ceretta AP, Peroza LR, de Moraes Reis E, Krum BN, et al. Harpagophytum Procumbens Ethyl Acetate Fraction Reduces Fluphenazine-Induced Vacuous Chewing Movements and Oxidative Stress in Rat Brain. Neurochem Res. 2016;41(5):1170-84.

51. Ucuncu Y, Celik N, Ozturk C, Turkoglu M, Cetin N, Kockara N, et al. Chondroprotective effects of a new glucosamine combination in rats: Gene expression, biochemical and histopathological evaluation. Life Sci. 2015;130:31-7.

52. Bastaki SMA, Amir N, Adeghate E, Ojha S. Lycopodium Mitigates Oxidative Stress and Inflammation in the Colonic Mucosa of Acetic Acid-Induced Colitis in Rats. Molecules. 2022;27(9).

53. Kim JY, Park SH, Baek JM, Erkhembaatar M, Kim MS, Yoon KH, et al. Harpagoside Inhibits RANKL-Induced Osteoclastogenesis via Syk-Btk-PLCγ2-Ca(2+) Signaling Pathway and Prevents Inflammation-Mediated Bone Loss. J Nat Prod. 2015;78(9):2167-74.

54. Chung HJ, Kyung Kim W, Joo Park H, Cho L, Kim MR, Kim MJ, et al. Anti-osteoporotic activity of harpagide by regulation of bone formation in osteoblast cell culture and ovariectomy-induced bone loss mouse models. Journal of ethnopharmacology. 2016;179:66-75.

55. Chung HJ, Kim WK, Oh J, Kim MR, Shin JS, Lee J, et al. Anti-Osteoporotic Activity of Harpagoside by Upregulation of the BMP2 and Wnt Signaling Pathways in Osteoblasts and Suppression of Differentiation in Osteoclasts. J Nat Prod. 2017;80(2):434-42.

56. Almeida MC, Soares SF, Abreu PR, Jesus LM, Brito LC, Bernardo-Filho M. Protective effect of an aqueous extract of Harpagophytum procumbens upon Escherichia coli strains submitted to the lethal action of stannous chloride. Cell Mol Biol (Noisy-le-grand). 2007;53 Suppl:Ol923-7.

57. Torres-Fuentes C, Theeuwes WF, McMullen MK, McMullen AK, Dinan TG, Cryan JF, et al. Devil's Claw to suppress appetite--ghrelin receptor modulation potential of a Harpagophytum procumbens root extract. PLoS One. 2014;9(7):e103118.

58. Laudahn D, Walper A. Efficacy and tolerance of Harpagophytum extract LI 174 in patients with chronic non-radicular back pain. Phytotherapy research : PTR. 2001;15(7):621-4.

59. Wegener T, Lüpke NP. Treatment of patients with arthrosis of hip or knee with an aqueous extract of devil's claw (Harpagophytum procumbens DC.). Phytotherapy research : PTR. 2003;17(10):1165-72.

60. Warnock M, McBean D, Suter A, Tan J, Whittaker P. Effectiveness and safety of Devil's Claw tablets in patients with general rheumatic disorders. Phytotherapy research : PTR. 2007;21(12):1228-33.

61. Puigdellivol J, Comellas Berenger C, Pérez Fernández M, Cowalinsky Millán JM, Carreras Vidal C, Gil Gil I, et al. Effectiveness of a Dietary Supplement Containing Hydrolyzed Collagen, Chondroitin Sulfate, and Glucosamine in Pain Reduction and Functional Capacity in Osteoarthritis Patients. J Diet Suppl. 2019;16(4):379-89.

62. Żęgota Z, Goździk J, Głogowska-Szeląg J. Prospective, Multicenter Evaluation of a Polyherbal Supplement alongside Standard-of-Care Treatment for Mild Knee Osteoarthritis. Adv Orthop. 2021;2021:5589597.

63. Puigdellívol Grifell J, Comellas Berenguer C, Steinbacher G, Kranjcec T, Álvarez Díaz P, López Pujol A, et al. Open, Observational, Single-Arm, Multicenter Study Assessing the Effectiveness of a Dietary Supplement Containing Hydrolyzed Collagen, Chondroitin Sulfate, and Glucosamine for Osteoarthritis Pain Reduction. J Diet Suppl. 2024;21(3):374-88.
